# The fall, recovery, classification, and initial characterization of the Hamburg, Michigan H4 chondrite

**DOI:** 10.1111/maps.13584

**Published:** 2020-10-27

**Authors:** Philipp R. Heck, Jennika Greer, Joseph S. Boesenberg, Audrey Bouvier, Marc W. Caffee, William S. Cassata, Catherine Corrigan, Andrew M. Davis, Donald W. Davis, Marc Fries, Mike Hankey, Peter Jenniskens, Philippe Schmitt‐Kopplin, Shannon Sheu, Reto Trappitsch, Michael Velbel, Brandon Weller, Kees Welten, Qing‐Zhu Yin, Matthew E. Sanborn, Karen Ziegler, Douglas Rowland, Kenneth L. Verosub, Qin Zhou, Yu Liu, Guoqiang Tang, Qiuli Li, Xianhua Li, Zoltan Zajacz

**Affiliations:** ^1^ Robert A. Pritzker Center for Meteoritics and Polar Studies Negaunee Integrative Research Center The Field Museum of Natural History 1400 South Lake Shore Drive Chicago Illinois 60605 USA; ^2^ Chicago Center for Cosmochemistry and Department of the Geophysical Sciences The University of Chicago 5734 South Ellis Avenue Chicago Illinois 60637‐1433 USA; ^3^ Department of Earth, Environmental and Planetary Sciences Brown University 324 Brook Street Box 1846 Providence Rhode Islands 02912 USA; ^4^ Bayerisches Geoinstitut Universität Bayreuth Universitätsstraße 30 95447 Bayreuth Germany; ^5^ Department of Earth Sciences University of Western Ontario BGS 1026, 1151 Richmond Street London Ontario N6A 5B7 Canada; ^6^ Department of Physics and Astronomy Purdue University West Lafayette Indiana 47906 USA; ^7^ Department of Earth, Atmospheric, and Planetary Sciences Purdue University West Lafayette Indiana 47906 USA; ^8^ Nuclear and Chemical Sciences Division Lawrence Livermore National Laboratory 7000 East Avenue (L‐235) Livermore California 94550 USA; ^9^ Department of Mineral Sciences National Museum of Natural History Smithsonian Institution 10th St and Constitution Ave, NW Washington District of Columbia USA; ^10^ Enrico Fermi Institute The University of Chicago 5734 South Ellis Avenue Chicago Illinois 60637‐1433 USA; ^11^ Department of Earth Sciences University of Toronto 22 Russell St Toronto Ontario M5S 3B1 Canada; ^12^ Astromaterials Research and Exploration Science Division NASA Johnson Space Center Mail Code XI2 Building 31 Houston Texas USA; ^13^ American Meteor Society 54 Westview Crescent Geneseo New York 14454 USA; ^14^ SETI Institute 189 Bernardo Avenue Mountain View California 94043 USA; ^15^ NASA Ames Research Center Moffett Field California 94035 USA; ^16^ Helmholtz Zentrum München Deutsches Forschungszentrum für Gesundheit und Umwelt (GmbH), Ingolstädter Landstr. 1 85764 Neuherberg Germany; ^17^ Department of Earth and Environmental Sciences Michigan State University 288 Farm Lane, 207 Natural Sciences Building East Lansing Michigan 48824 USA; ^18^ Albany Medical College 43 New Scotland Ave Albany New York 12208 USA; ^19^ Space Sciences Laboratory University of California 7 Gauss Way Berkeley California 94720‐7450 USA; ^20^ Department of Earth and Planetary Sciences University of California at Davis One Shields Avenue Davis California 95616 USA; ^21^ Institute of Meteoritics University of New Mexico 221 Yale Blvd NE, 313 Northrop Hall Albuquerque New Mexico 87131 USA; ^22^ Center for Molecular and Genomic Imaging University of California at Davis Davis California 95616 USA; ^23^ National Astronomical Observatories Chinese Academy of Sciences Beijing 100012 China; ^24^ State Key Laboratory of Lithospheric Evolution Institute of Geology and Geophysics Chinese Academy of Sciences Beijing 100029 China

## Abstract

The Hamburg meteorite fell on January 16, 2018, near Hamburg, Michigan, after a fireball event widely observed in the U.S. Midwest and in Ontario, Canada. Several fragments fell onto frozen surfaces of lakes and, thanks to weather radar data, were recovered days after the fall. The studied rock fragments show no or little signs of terrestrial weathering. Here, we present the initial results from an international consortium study to describe the fall, characterize the meteorite, and probe the collision history of Hamburg. About 1 kg of recovered meteorites was initially reported. Petrology, mineral chemistry, trace element and organic chemistry, and O and Cr isotopic compositions are characteristic of H4 chondrites. Cosmic ray exposure ages based on cosmogenic ^3^He, ^21^Ne, and ^38^Ar are ~12 Ma, and roughly agree with each other. Noble gas data as well as the cosmogenic ^10^Be concentration point to a small 40–60 cm diameter meteoroid. An ^40^Ar‐^39^Ar age of 4532 ± 24 Ma indicates no major impact event occurring later in its evolutionary history, consistent with data of other H4 chondrites. Microanalyses of phosphates with LA‐ICPMS give an average Pb‐Pb age of 4549 ± 36 Ma. This is in good agreement with the average SIMS Pb‐Pb phosphate age of 4535.3 ± 9.5 Ma and U‐Pb Concordia age of 4535 ± 10 Ma. The weighted average age of 4541.6 ± 9.5 Ma reflects the metamorphic phosphate crystallization age after parent body formation in the early solar system.

## Introduction

**Fig. 1:**
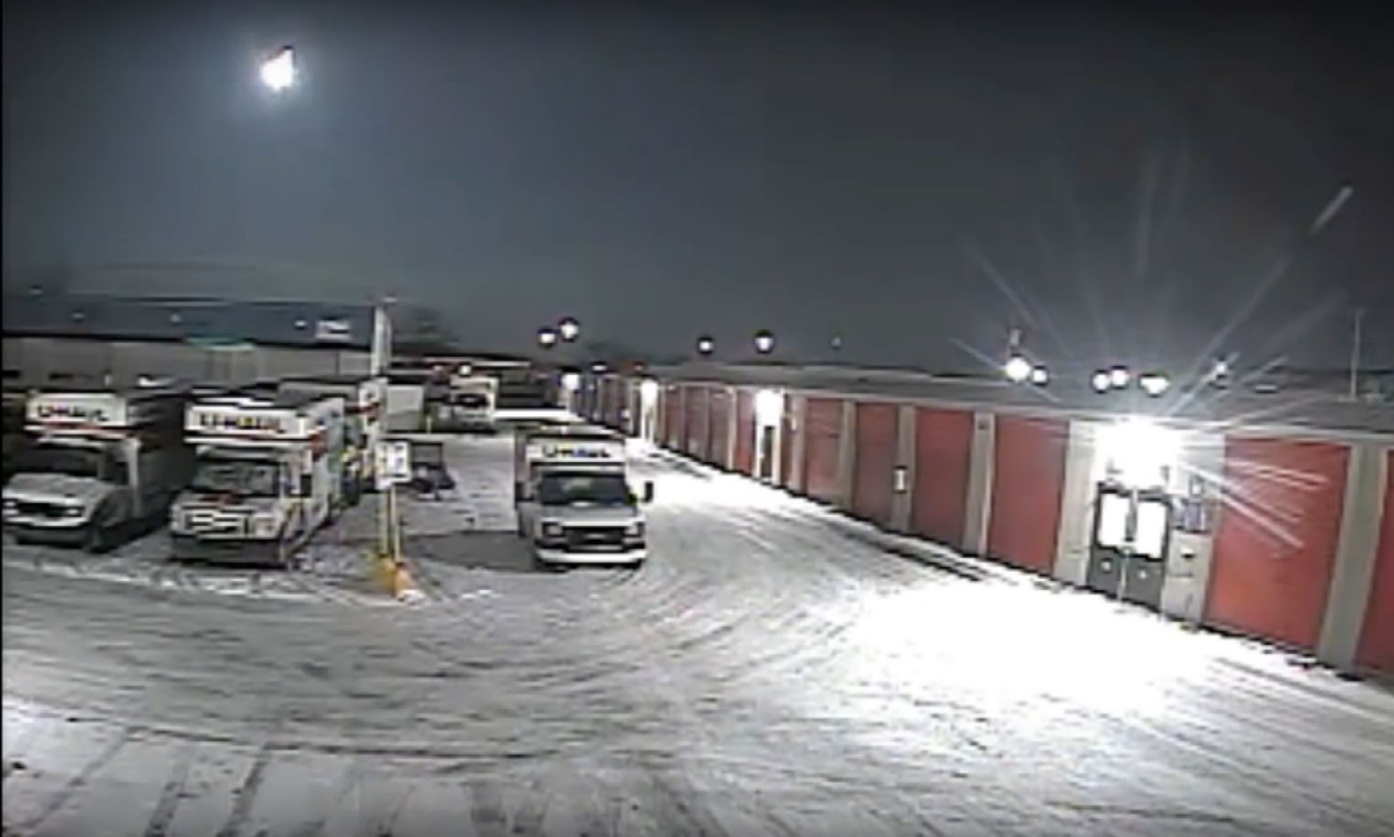
Still frame from security video of the Hamburg fireball recorded from Toledo OH. Image credit: T. Masterson. The video is available to watch on the American Meteor Society Website. AMS Event: 168‐2018, Report 135170 (168nd‐2018). (Color figure can be viewed at wileyonlinelibrary.com.)

On January 16, 2018 at 01:08 UTC (January 16, 2018, 20:08 EST local time), a fireball was observed and reported by 674 witnesses from 10 U.S. states (Michigan, Illinois, Wisconsin, Ohio, Pennsylvania, Indiana, West Virginia, Georgia, Missouri, and Kentucky) and Ontario, Canada, to the American Meteor Society (AMS event #2018‐168). Footage of the fireball was acquired by multiple security cameras (e.g., Fig. 1), from which a trajectory and pre‐impact orbit were later published (Brown et al. 2019). The atmospheric shockwave was registered by several infrasound sensors in the Central and Eastern United States and six seismometer stations as the equivalent of a 1.8 ± 0.2 magnitude earthquake (source: https://prod‐earthquake.cr.usgs.gov/earthquakes/eventpage/us2000ck7p/origin/magnitude; Bormann and Dewey 2012; Hedlin et al. 2018).

**Fig. 2:**
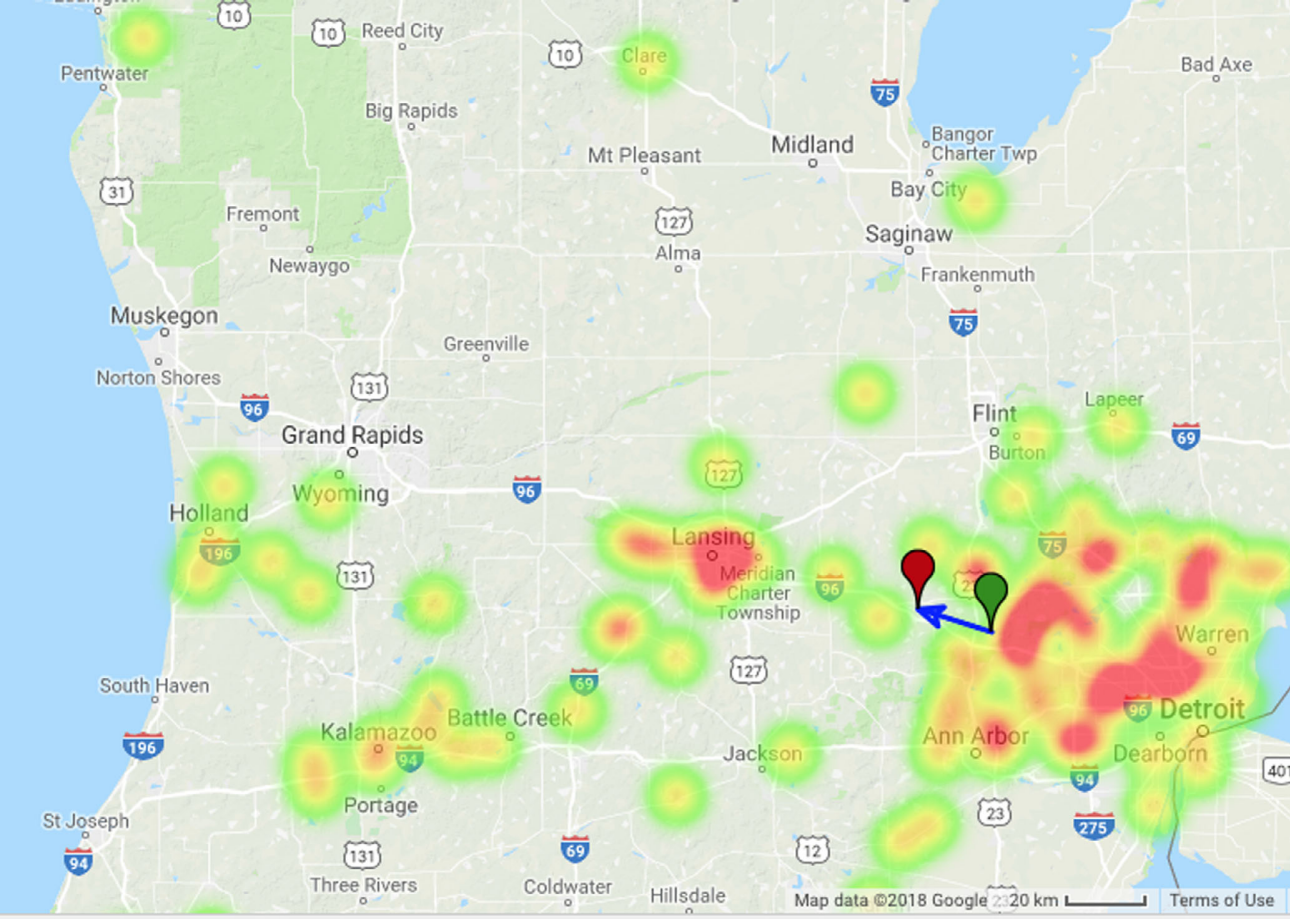
Density “heat map” of the intensity of reported fireball observations and calculated fireball trajectory. Green and red markers show the start and the end of the observed fireball, respectively. Courtesy American Meteor Society. https://www.amsmeteors.org/2018/01/bright‐fireball‐spotted‐overmichigan/. (Color figure can be viewed at wileyonlinelibrary.com.)

**Fig. 3:**
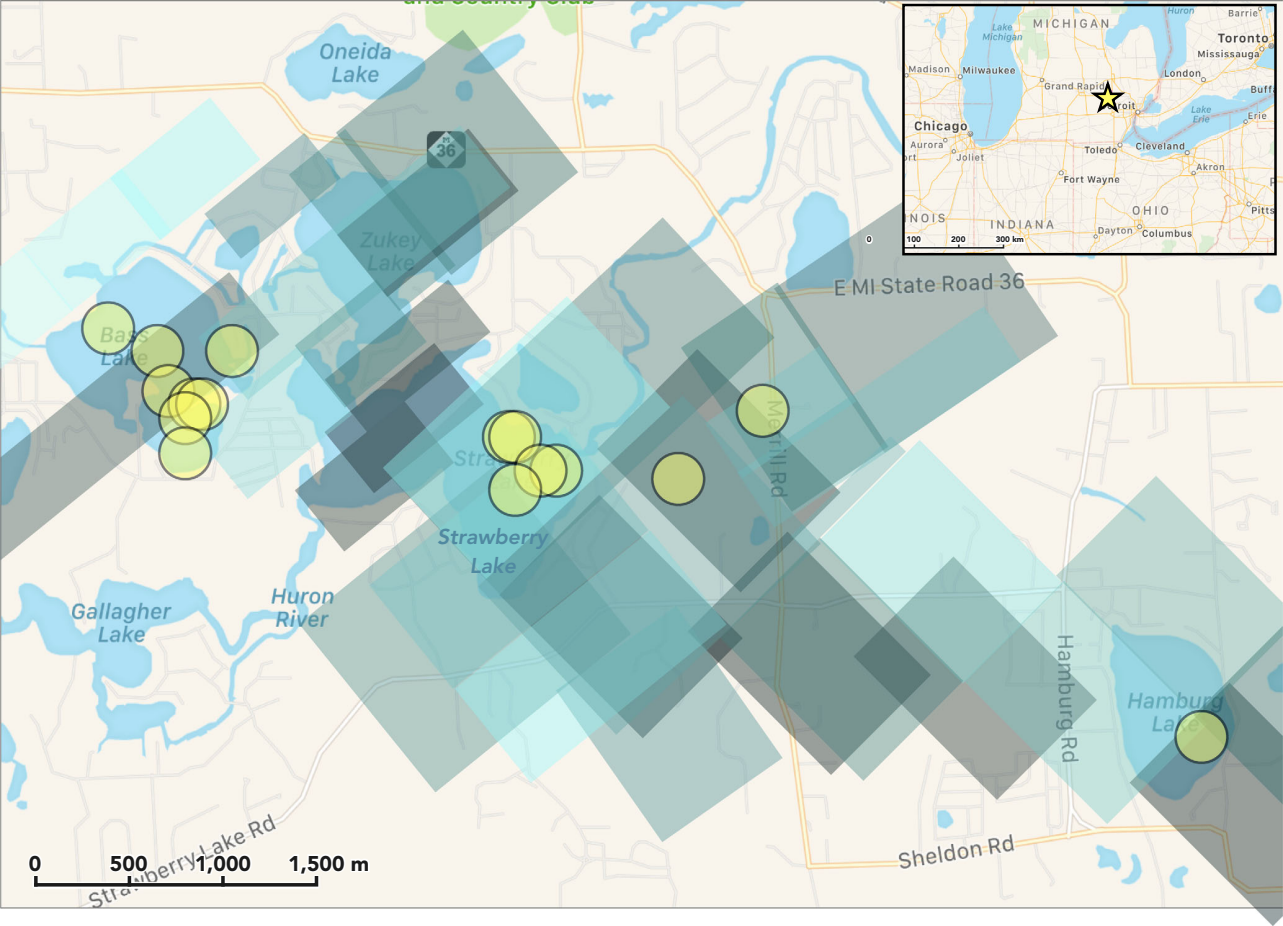
Compilation of all radar signatures from the Hamburg meteorite fall (gray/blue polygons at center), describing an elongate signature of falling meteorites. The radar pixels represent reflectivity (with higher reflectivity shown as darker pixels), or a measure of radar energy returned to the radar off of falling meteorites. The fireball traveled approximately east to west, and the resulting size range of meteorites generally trends from larger meteorites at the west grading toward smaller meteorites toward the eastern end of the radar signatures. In this strewn field map, the 16 first Hamburg meteorites found within 2 weeks of the fall are shown as circles. Coordinates of fall: 42°26.82′N, 83°50.5′W (positions from American Meteor Society; image credit: Apple Maps). (Color figure can be viewed at wileyonlinelibrary.com.)

Visual observations were used to calculate an approximate trajectory (Fig. 2, Table 1), which was used to identify the signature of falling meteorites in Doppler weather radar reflections. From this, a strewn field map was prepared using weather radar data from radar reflections of falling meteorites (Fig. 3), which guided meteorite hunters with their recovery efforts.

**Table 1:** Appearance time and altitude for the radar detection of falling meteorites.

Radar	Elevation (°)	Est. mass (g)	Refl ASL (m)	Time of detection (UTC)
KDTX 0107	2.5	4.97	2234	1:13:03
KDTX 0107	3.5	0.295	2820	1:15:27
TDTW 0115	0.6	0.5035	813	1:16:21
TDTW 0115	0.1	0.4885	387	1:16:35
KDTX 0107	4.5	0.076	3505	1:16:40
TDTW 0121	0.6	0.0173	743	1:22:12
TDTW 0139	1	(dust)	1052	1:40:58

Selection criteria for these radar signatures include sudden appearance at the time and place identified by eyewitness accounts; a general pattern of vertical (falling) motion as opposed to the horizontal motion of clouds and other radar targets (e.g., birds, aircraft); and pairing with Doppler turbulence signatures for the earlier appearing, more massive meteorites. Mass estimates are produced from the times and altitudes of the fireball terminus and individual radar returns, by calculating the size of meteorites that must travel through a three‐dimensional path between the terminus and the radar return in the time difference between the two events.

The first specimen, a complete individual, was found on January 18, 2018 at 7:50 EST by Robert Ward* *in the snow on the frozen surface of Strawberry Lake near Hamburg, MI. Five other masses, ranging from 17 to 102.6 g, were found later the same day by Ward, Larry Atkins, and Darryl Landry on Strawberry and Bass Lakes. Thirteen additional pieces were found within 2 weeks of the fall (Fig. 3).

**Fig. 4:**
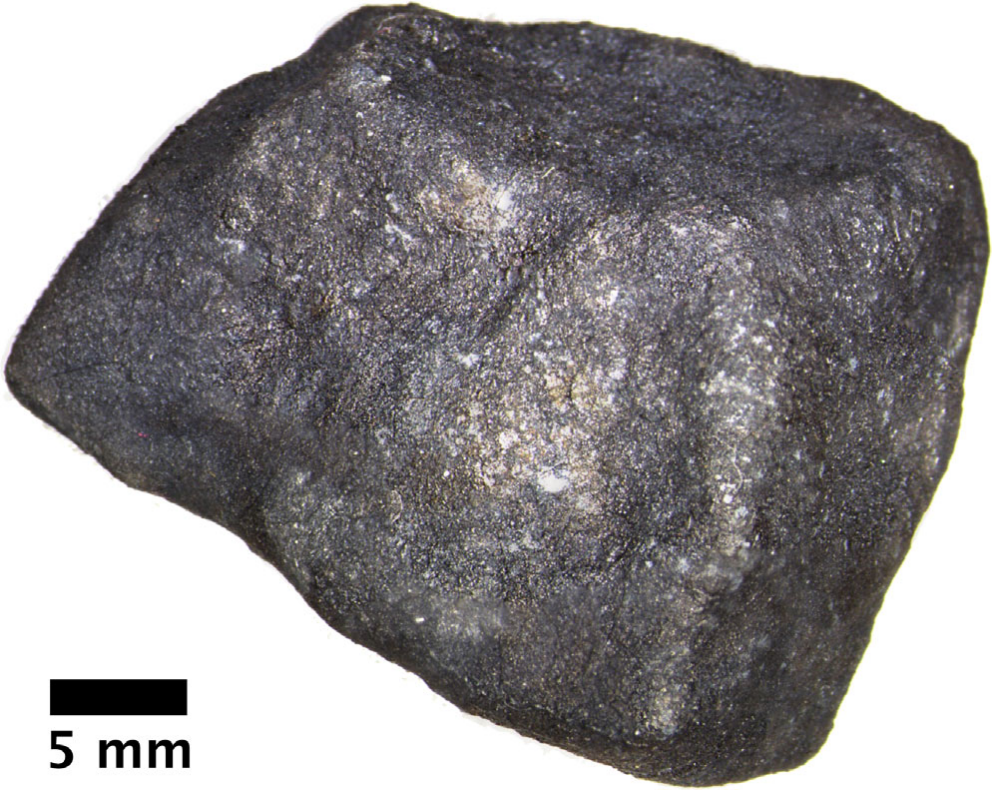
Optical micrograph of the complete Strawberry Lake individual of Hamburg ME 6108 before cutting. (Color figure can be viewed at wileyonlinelibrary.com.)

The first piece, found less than 2 days after the fall, was donated to the Field Museum by Ward and received on January 24, 2018. It was classified at the Field Museum and the University of Chicago as an H4 chondrite (Gattacceca et al. 2020). The Strawberry Lake type specimen of Hamburg, with a mass of 22.8 g (Fig. 4), FMNH ME 6108.1, and a polished thick section (FMNH ME 6108.3), are curated at the Field Museum’s Robert A. Pritzker Center for Meteoritics and Polar Studies meteorite collection. In addition, Atkins provided specimens from a 17 g stone recovered from Strawberry Lake, which is in a repository at Michigan State University (MSU Abrams Planetarium specimen 2018‐001; with the fall location recorded as AMS#24). A 59.4 g specimen was found on January 19, 2018, 3 days after the fall, also on Strawberry Lake by Brandon Weller.

Because these first specimens of the meteorite were rapidly recovered from an icy surface, the time period for terrestrial alteration was short; the meteorite has the potential to be preserved in a pristine condition. An international consortium was formed to thoroughly characterize the meteorite while it was still fresh.

Here, we present results of the studies performed shortly after the fall. The work presented here includes radar observations; mineralogical–petrological characterization and classification; µCT scan; bulk oxygen and chromium isotopic composition; trace element chemistry; organic chemistry; magnetic susceptibility; U‐Pb, Pb‐Pb, U‐Th/He, and Ar‐Ar geochronology; and noble gas cosmic ray exposure (CRE) age dating. A detailed description on the established methods used for this study can be found in the supporting information.

## Results and Discussion

### Weather Radar Observations and Calculations

Reflections from falling meteorites appear in data from two weather radars; an NOAA WSR‐88D weather radar sited in Detroit, MI, and a terminal Doppler weather radar (TDWR) serving the Detroit Metropolitan Airport. The first appearance of falling meteorites on radar occurs at 01:13:03 UTC and 2234 m above sea level (ASL) in the 0107 UTC data set for the KDTX radar in the 2.5 degree elevation radar sweep. Signatures consistent with falling meteorites appear in a total of seven radar sweeps from the two radars, with a small final signature appearing at 01:40:58 UTC (Fig. 3, Tables 1 and S1 in supporting information).

Based on the time it takes various masses to fall to the altitude of detection, and using an estimated fireball terminus altitude of 20 km and at the time 01:08:33 UTC derived from video of the event, masses calculated from radar data range from 4.97 g down to 0.0173 g, with the final detection composed of dust that is too light to accurately calculate. The actual range of masses present in the meteorite fall is much wider than this, but weather radar detection is most sensitive in the ~0.1 to 10 g range due to a combination of timing and detection sensitivity factors.

The total fallen mass of Hamburg meteorites is ~2 kg, based on a measure of the definite integral of the mass distribution over the mass range of 0.1–10 g, where the radar is the most sensitive. This is only about ~1% that of the Park Forest meteorite fall (Figs. S1 and S2 in supporting information). The overall mass distribution of Hamburg is at the lower end of the range observed for other ordinary chondrites (Fig. S2). Larger masses (>1 kg) may be present, but were not directly detected, so their inferred presence is based on modeling, which currently still contains large uncertainties. This value should be considered a preliminary estimate, as the methodology for meteorite mass estimation from radar data is currently in development and estimated mass errors could be as high as a factor of ~5.

The implied recovery ratio of ~50% of the radar‐detected meteorite seems high. However, the terrain in the Hamburg strewn field consisted of frozen lakes and snow cover that favored recovery.

### Magnetic Properties of Hamburg

Magnetic analyses can be compromised if specimens are placed close to strong artificial magnetic fields such as hand magnets (Gattacceca and Rochette 2004). One of the first analyses performed was the determination of the magnetic properties of Hamburg to see if this specimen could be used to shed light on the magnetic field in the solar nebula at the time of its formation. Because the sample was discoid in shape and because it did not fill the measurement volume of the susceptibility bridge, the sample was measured several times in two orientations: with the short axis vertical and with the short axis horizontal.

For the short axis vertical measurements, the log of the mean value of the magnetic susceptibility, in units of 10^–9^ m^3^ kg^–1^, (log χ) was 4.91. For the short axis horizontal measurements, the value of log χ was 5.12. A good estimate for the value of log χ for the specimen is 5.0, which is at the lower limit of the range of values reported for Type LL chondrites and the upper limit of the range of values reported for Type H chondrites (Rochette et al. 2003). No significant frequency‐dependent susceptibility was detected for this sample.

The sample was then subjected to alternating field demagnetization at levels of 0, 5, 10, 15, 20, 25, 30, 35, 40, 50, 60, 80, and 100 mT. The Hamburg sample we measured was found to have a moderately strong magnetization. After removal of a secondary component during the first two demagnetization steps, the sample exhibited a stable direction of magnetization with an intensity of 4.44 × 10^–3^ Am^2^ kg^–1^ after the 10 mT demagnetization step. This intensity was systematically reduced during demagnetization with less than 10% remaining after the 100 mT step. This behavior suggests we are looking at primordial magnetization.

To obtain an estimate of the ancient magnetic field in which the Hamburg had formed, it is necessary to determine the isothermal remanent magnetization (IRM) of the sample (Gattacceca and Rochette 2004; Acton et al. 2007). A progressive IRM acquisition experiment could be performed without saturating the detectors of the cryogenic magnetometer, even after application of an IRM‐inducing field of 10 T. However, the progressive alternating field demagnetization of this IRM produced data that behaved differently than the progressive alternating field demagnetization of the natural remanent magnetization (NRM). In the case of the IRM, this intensity was reduced during demagnetization to only 50% of its original value after the 100 mT step.

Three different values are commonly used to obtain the paleofield (Gattacceca and Rochette 2004; Acton et al. 2007). The first is called REM and is equal to the original NRM of a stone divided by the IRM acquired by the stone in a field of 10 T, before any demagnetization. The second method for obtaining the paleofield is REMc, which is the ratio of the NRM to IRM after each magnetization has been subjected to a low demagnetizing field, typically 20 mT (the “c” stands for the coercivity spectrum representative of the characteristic remanent magnetization; Acton et al. 2007). The third measurement, designated REM´, is the ratio of the change in NRM divided by the change in IRM over a specific demagnetization interval. The rule of thumb is that the NRM/IRM ratio times 3000 gives the paleofield (Gattacceca and Rochette 2004; Kletetschka et al. 2004).

Because the Hamburg sample exhibited a secondary NRM component, direct calculation of REM was deemed to be inappropriate. However, from the behavior of the NRM at higher demagnetization levels, it was possible to extrapolate the NRM to its initial (unremagnetized) value. Using this approach, we obtain a REM value of 0.354.

Over the demagnetization interval from 20 to 60 mT, the NRM/IRM ratio at specific demagnetization levels (REMc) ranged from 0.288 to 0.115. The disparity between these values is not surprising, given that during demagnetization the NRM loses its magnetization at a faster rate than the IRM. Similarly, the value of REM´ for the demagnetization range 20–60 mT is 1.39, which simply reflects the fact that a greater percentage of the NRM intensity is lost during demagnetization over this range than IRM intensity is lost. These discrepancies illustrate the difficulties of trying to use Hamburg to determine a paleofield value.

All of the NRM/IRM paleofield methods depend on the assumption that the NRM and the IRM are carried by the same population of magnetic grains. The difference between the NRM and IRM behavior suggests that the IRM acquisition process activated a higher coercivity population than was present when the NRM magnetization was formed. The most likely explanation for this is that subsequent metamorphism has transformed some of the low‐coercivity magnetic fraction into a high‐coercivity magnetic fraction.

### Petrology and Mineralogy

**Fig. 5:**
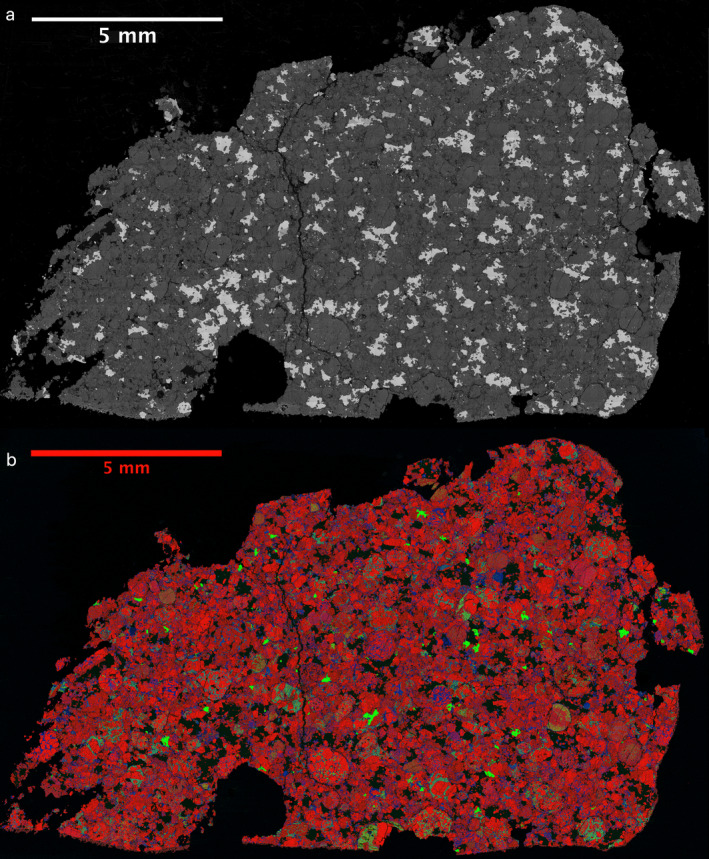
a) Backscattered electron image and (b) composite RGB EDS map of the “Strawberry Lake” Hamburg type specimen section ME 6108.3. Red = Mg; green = Ca; blue; Al. (Color figure can be viewed at wileyonlinelibrary.com.)

The next step in the process was characterizing the petrology and mineralogy of the meteorite. Most of the recovered stones of Hamburg are fully covered by fusion crust. All specimens show the same texture with chondrules obvious on polished surfaces. The metal‐rich texture typical of H chondrites is apparent on cut surfaces (Fig. 5a), and most chondrules have sharp boundaries, consistent with petrologic type 4 chondrites. The description below is based on microscopy of sections of the Field Museum type specimen (ME 6108.3), the Michigan State University Abrams Planetarium specimen (2018‐001a‐TS), and the section studied at Brown University.

**Fig. 6:**
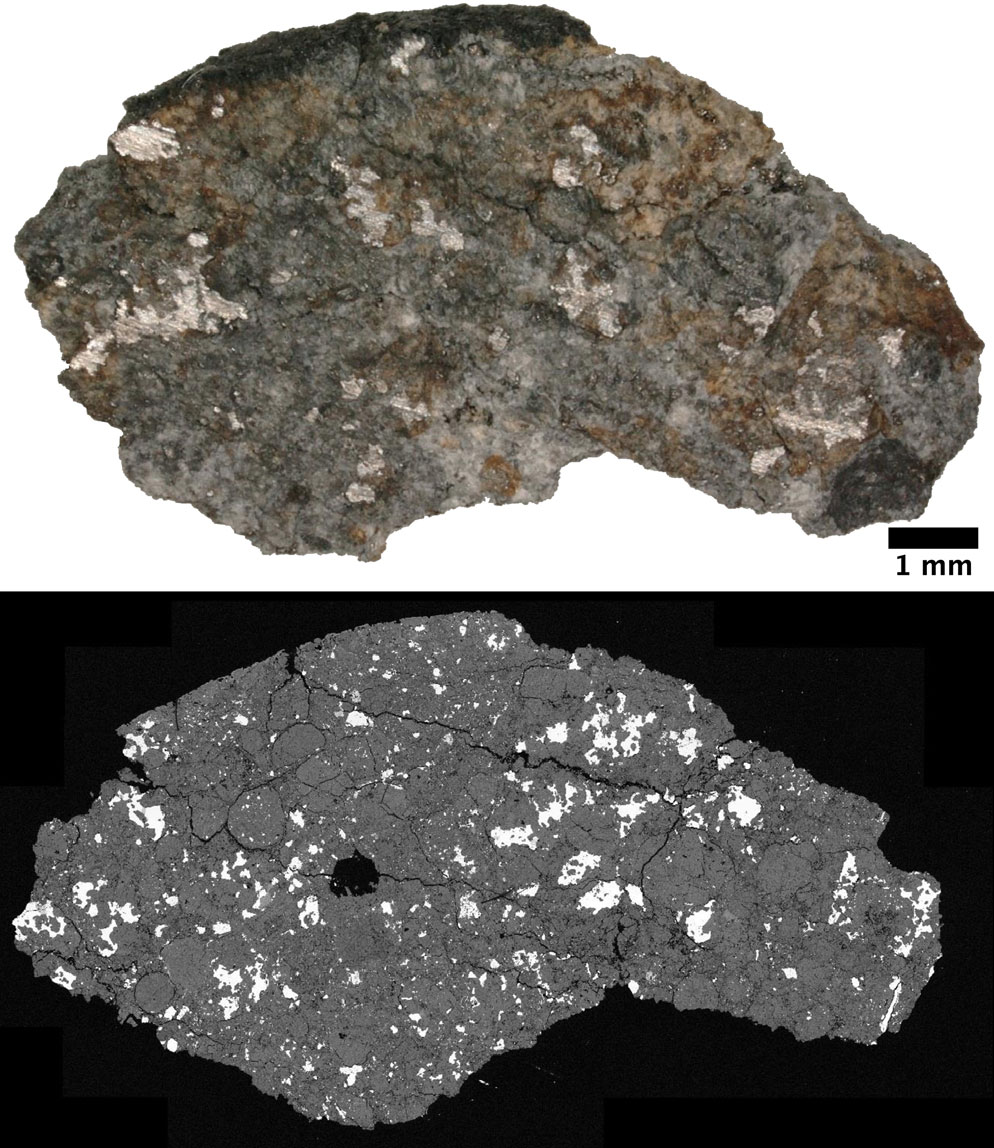
Top: Before thin sectioning, the area exposed as a gently curved cashew‐shaped outline was 10 mm in longest dimension, and the maximum dimension perpendicular to the length was 5 mm. About 4 mm of the long convex edge of this sectioned piece preserved a thin (sub‐mm) fusion crust. Extended focus reflected visible light image of the slice of MSU Hamburg 2018‐001 from which the thin section was prepared. Some staining from weathering is visible in this specimen. Bottom: Backscattered electron microscope image of MSU thin section 2018‐001a‐TS. Same scale for both images. (Color figure can be viewed at wileyonlinelibrary.com.)

The type specimen is an individual almost fully covered with fusion crust, with only a few mm^2^ missing, which enabled a view of the interior before cutting. Raman spectroscopy of exposed olivine crystals enabled an initial H chondrite classification before cutting, using the method described by Kuebler et al. (2006). No visual indication of weathering was observed on the type specimen, which was the first sample to have been recovered from the fall. The MSU‐Abrams sections and the section studied at Brown University show some staining (Fig. 6a). Matrix, some rusty (possibly due to early onset of terrestrial weathering [Velbel 2014] of these specimens), dominated the exposed surfaces, and metal was distributed irregularly throughout. On the polished cross section of the type specimen, the metal volume estimate is 9% based on an Fe‐Ni EDS map. The metal has a composition of 6.6 wt% Ni and 0.55 wt% Co with P, Cr, Mn, and S below detection limits.

**Fig. 7:**
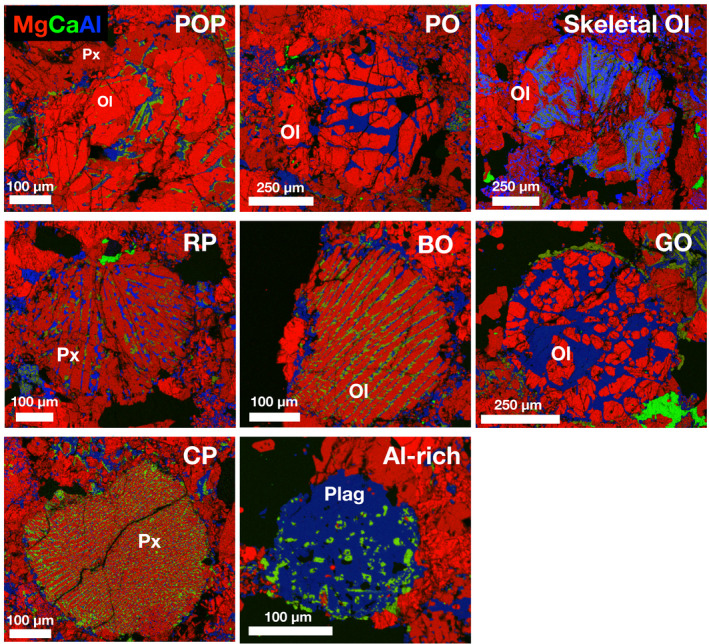
Composite RGB EDS maps of different types of chondrules found in the Hamburg type specimen section. Abbreviations: POP = porphyritic olivine with pyroxene; porphyritic olivine (PO), Ol = olivine; Px = pyroxene; radial pyroxene (RP); barred olivine (BO); cryptocrystalline (C) chondrules with pyroxene, granular olivine (GO). Red = Mg; green = Ca; blue = Al. (Color figure can be viewed at wileyonlinelibrary.com.)

**Fig. 8:**
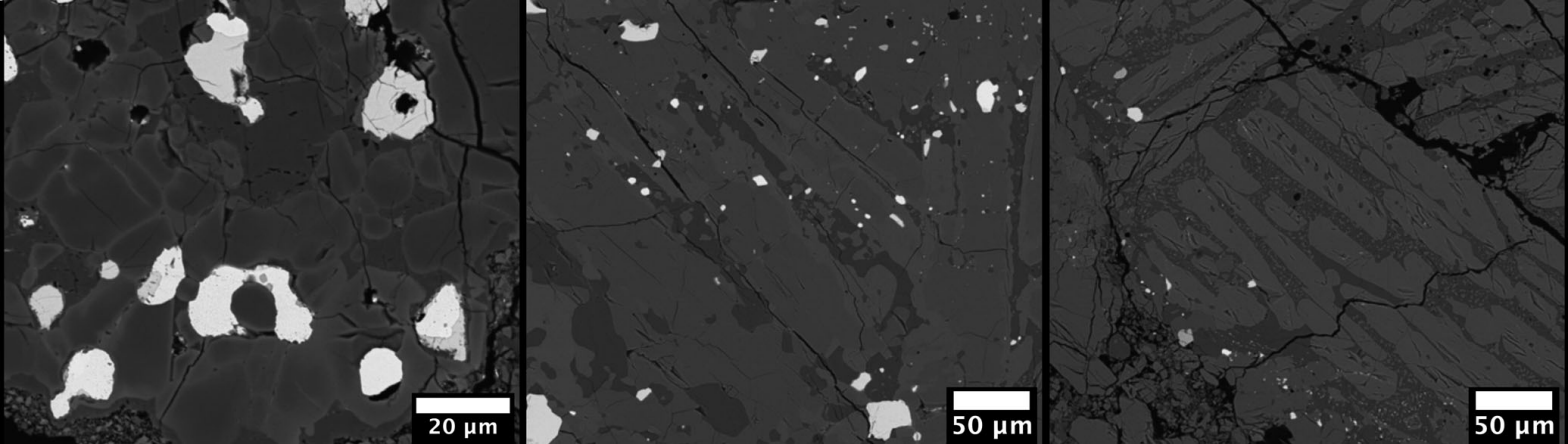
Chondrules from the MSU section Hamburg 2018‐001a‐TS. Left: Close‐up of porphyritic olivine–pyroxene chondrule with zoned olivine and zoned low‐Ca pyroxene zoning. Middle: Porphyritic pyroxene chondrule with slightly zoned low‐Ca pyroxene; some high‐Ca pyroxene also occurs in this chondrule. Right: Close‐up of a barred olivine chondrule. Several olivine bars show elongated growth habit with centrally located glass inclusions.

Chondrules, often with sharp boundaries, are conspicuous. The average apparent chondrule diameter was determined to be 0.44 ± 0.21 SD ± 0.02 SE mm (*n* = 144; SD = 1 standard deviation, SE = standard error), consistent with the average H chondrite apparent chondrule diameter (0.3 mm; Rubin 2000). Chondrules are diverse and well defined; several examples are shown in Figs. 7 and 8. Porphyritic olivine chondrules with some pyroxene (POP) and porphyritic olivine chondrules (PO) dominate. This is followed by radial pyroxene (RP) and barred olivine (BO) chondrules. A few cryptocrystalline (CP) chondrules with pyroxene, granular olivine (GO), skeletal olivine chondrules, and aluminum‐rich plagioclase chondrules were also observed in the type specimen. In the MSU‐Abrams specimen, several less common textures are present—porphyritic pyroxene (PP, *n* = 1), PO with BO inclusions (*n* = 1), and BO with PP glomerocrysts (*n* = 1). Microphenocrysts in some porphyritic chondrules have oriented skeletal outgrowths. In the Brown University section, the only unusual chondrule was one porphyritic olivine chondrule containing Fo_99_ olivine.

Olivine compositions are homogeneous within individual crystals and among different crystals and averaged to Fa_18.7±0.7 _(*n* = 34; 1SD) in the type specimen and Fa_17.0±3.9_ (*N* = 89; 1SD) in the MSU‐Abrams specimen. Olivine bars in all barred olivine chondrules (BO, hybrid BO, compound PO‐hosting BO) are compositionally uniform throughout (Fa_18.1–18.9_, *n* = 39). Olivine and low‐Ca pyroxene microphenocrysts exhibit at least slight normal zoning (apparent with elevated Z‐contrast) near edges in all porphyritic chondrules in which olivine and pyroxene co‐occur (POP, PO; Figs. 8A and 8B). The visible zoning is spatially associated with compositional variation as measured by EPMA (Fa_4.0–26.3_, *n* = 36; Fs_0.1–20.2_, *n* = 95). The outermost zones of olivine co‐occurring with low‐Ca pyroxene in these chondrules are substantially more iron‐rich (up to Fa_26.3_) than the homogenous compositions of barred olivine (all Fa_18.1–18.9_ (*n* = 39 analyses from *n* = 6 chondrules). The outermost zones of low‐Ca pyroxene all fall within a narrow range (Fs_15.9–20.2_, *n* = 95 analyses from *n* = 4 chondrules). The RGB map of the skeletal olivine PO chondrule in the FMNH type specimen (Fig. 7, top row right) shows the same compositional zoning as is shown in the BSE images of the MSU Abrams Planetarium sample (Figs. 8A and 8B) and the corresponding EPMA data. This suggests that Hamburg is homogenous with respect to the occurrence and distribution of zoned olivine and pyroxene.

Both porphyritic and barred olivine chondrules show textural and compositional evidence of disequilibrium during rapid cooling. Skeletal (terminology of Drever and Johnston 1957; Fleet 1975; Kirkpatrick 1975) or hopper (terminology of Donaldson 1976) overgrowths/outgrowths on olivine phenocrysts indicate moderately rapid (disequilibrium) cooling at modest supercooling near the end of chondrule cooling (e.g., Donaldson 1976). Olivine bars exhibiting elongated growth habits with centrally located glass inclusions (Fig. 8C; terminology of Drever and Johnston [1957], also illustrated by Donaldson [1976]) occur sparsely but are widely distributed; they indicate faster cooling rates at higher degrees of supercooling (Donaldson 1976). Backscattered electron imaging and X‐ray spectroscopy observations suggest the presence of glass and some devitrification in several chondrules.

Pyroxenes are mostly orthopyroxene and less abundant clinopyroxene as determined with Raman spectroscopy. The average composition of Ca‐poor pyroxene is Fs_16.3±0.4_Wo_1.3±0.1 _(*n* = 80; 1SD) in the type specimen and Fs_15.8±2.7_Wo_1.4±0.3_ (*n* = 85) in the MSU‐Abrams specimen. Feldspar of various sizes was observed in the meteorite and measured, on average, 3.4 ± 2.2 μm (*n* = 64; 1SD) in the longest dimension within representative fields of views (e.g., Fig. S3 in supporting information). Feldspar in the type specimen has an average composition of An_14.0±4.0_Ab_81.1±3.0 _Or_4.8±1.3 _(*n* = 13).

Mean compositions of the cores of the major silicates fall within the H‐chondrite range, and the well‐defined textural distinction between chondrules and matrix corresponds to petrologic type 4.

Phosphates account for about 0.5% of the meteorite by volume and occur mainly as merrillite (0.4%) and apatite (0.1%). In a representative field of view, grain sizes for merrillite average around 100 µm and range from <1 µm up to 430 µm (*n* = 37); apatite ranges from 70 to 310 µm with an average of 150 µm (*n* = 8). Apatite contains about 5 wt% Cl and <1 wt% F. Chromites are impact fractured and neither chromite veinlets nor veins were observed within the meteorite. The average minor element composition of chromite is TiO_2_ = 2.0 ± 0.4 wt% and V_2_O_3_ = 0.8 ± 0.2 wt% (*n* = 25). A single ilmenite grain is present in the type specimen section. Melt veins (Fe‐sulfide) occur only in proximity to the fusion crust. Multiple cross‐cutting fractures are observed in all sections. Two observed primary fractures partially cross‐cutting the Brown University section both contain troilite. Unlike the melt veins observed in the type specimen, where the S‐rich melt veins only occurred near fusion crust as a result of atmospheric heating during entry, the mobilization in this specimen seems to have taken place on the parent body. These observations of fractures, together with the absence of other shock features, indicate a very weak shock stage (S2; Stöffler et al. 1991).

In summary, the petrology, mineral chemistry, and compositional heterogeneity of the minerals listed are consistent with H4 chondrites. The meteorite was classified as H4, S2, W0.

#### Computed Microtomography

Part of the petrological characterization for this study was computed microtomography. This method is particularly useful to detect different lithologies within a rock. The representative image and animation (Fig. S4 in supporting information) reveal several aspects regarding the petrography of this meteorite which are also seen in the SEM images. There is a rich background of chondrules interspersed throughout the fine‐grained matrix. Chondrules as small as 200 μm and as large as 2 mm could be seen within the sample. The Fe composites (FeNi metal has the brightest intensity and Fe sulfides are slightly darker than FeNi metal) have a high abundance consistent with H chondrites. Image intensity was adjusted to allow for visual differentiation of the FeNi and Fe‐sulfide inclusions.

### Oxygen and Chromium Isotopes

**Fig. 9:**
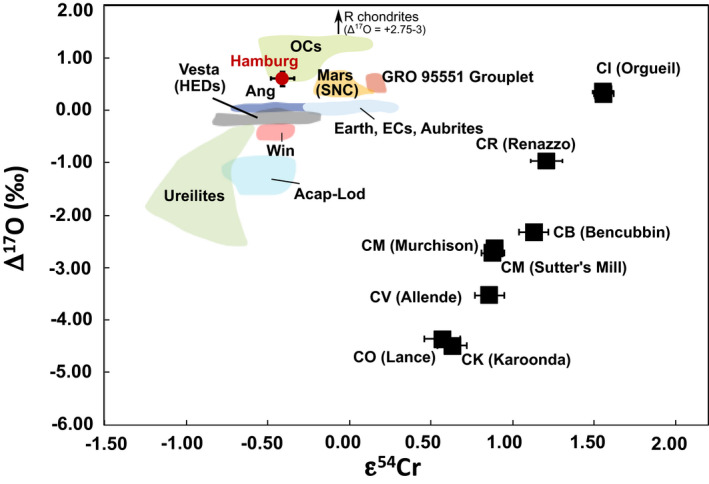
Oxygen and chromium isotopic composition of Hamburg. Literature data for Δ^17^O (Clayton et al. 1984, 1991; Clayton and Mayeda 1996, 1999; Scott et al. 2009; Jenniskens et al. 2012, 2014; Popova et al. 2013). Literature Cr data (Shukolyukov and Lugmair 2006; Ueda et al. 2006; Trinquier et al. 2007; Jenniskens et al. 2012, 2014; Popova et al. 2013; Sanborn and Yin 2014; Schmitz et al. 2016). (Color figure can be viewed at wileyonlinelibrary.com.)

After characterizing the petrology and mineralogy, we determined the chromium and oxygen isotopic composition of the rock, both standard methods for meteorite classification (e.g., Krot et al. 2014). Chromium isotopic measurements yield an ε^54^Cr value for the bulk Hamburg subsample of –0.41 ± 0.07 (Table 2). The deficit in the ^54^Cr/^52^Cr relative to terrestrial composition is typical of ordinary chondrites (ε^54^Cr = –0.19 ± 0.13 to –0.47 ± 0.07; Trinquier et al. 2007; Popova et al. 2013; Jenniskens et al. 2014). Oxygen isotopic analyses were made (see supporting information) in triplicate with values of δ^17^O′ = 2.715 ± 0.187 (1σ; *n* = 3) and δ^18^O′ = 4.036 ± 0.233 (1; *n* = 3). These values translate to an average mass independent Δ^17^O′ value of 0.585 ± 0.068 (1σ; *n* = 3). When combining the ε^54^Cr and Δ^17^O′ values, Hamburg plots in the lower end of the ordinary chondrite composition field (Fig. 9). The indistinguishable ε^54^Cr and Δ^17^O′ between Hamburg and the H chondrites further confirm its classification as an H chondrite.

**Table 2:** Oxygen and chromium isotopic composition of Hamburg.

	Mass (mg)	δ^17^O′ (±1σ)	δ^18^O′ (±1σ)	Δ^17^O′ (±1σ)	ε^54^Cr (±2SE)
	1.9	2.575	3.830	0.553	
	1.2	2.927	4.288	0.663	
	1.3	2.644	3.989	0.538	
Average		2.715 ± 0.187	4.036 ± 0.233	0.585 ± 0.068	−0.41 ± 0.07

### Trace Element Geochemistry of Phosphates

**Fig. 10:**
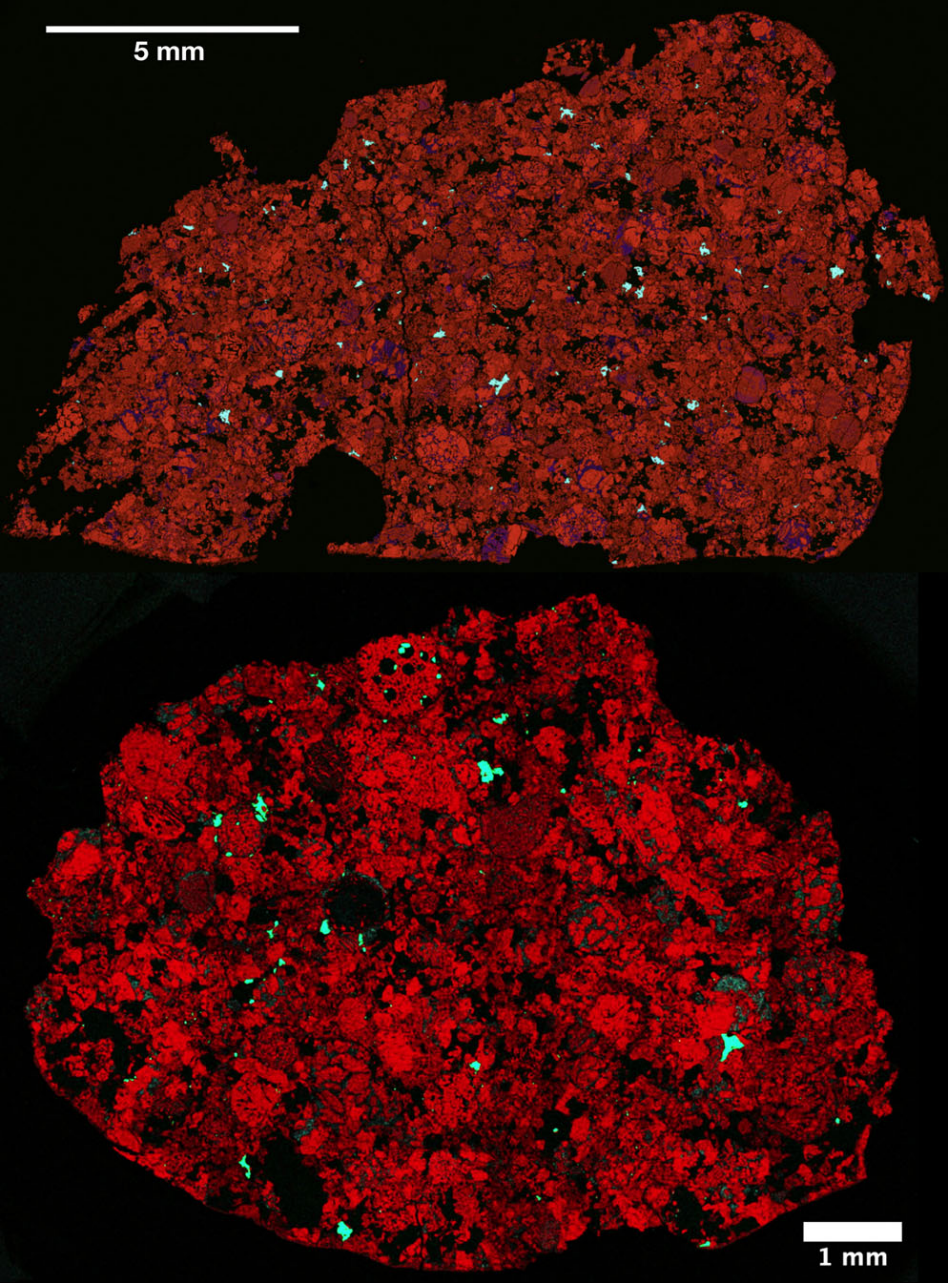
RGB map of phosphates from top: type specimen ME6108.3 studied by LA‐ICPMS, and bottom: specimen (aliquot from MSU‐Abrams 2018‐001) studied by SIMS (Mg—red, P—green, Ca—blue). (Color figure can be viewed at wileyonlinelibrary.com.)

**Fig. 11:**
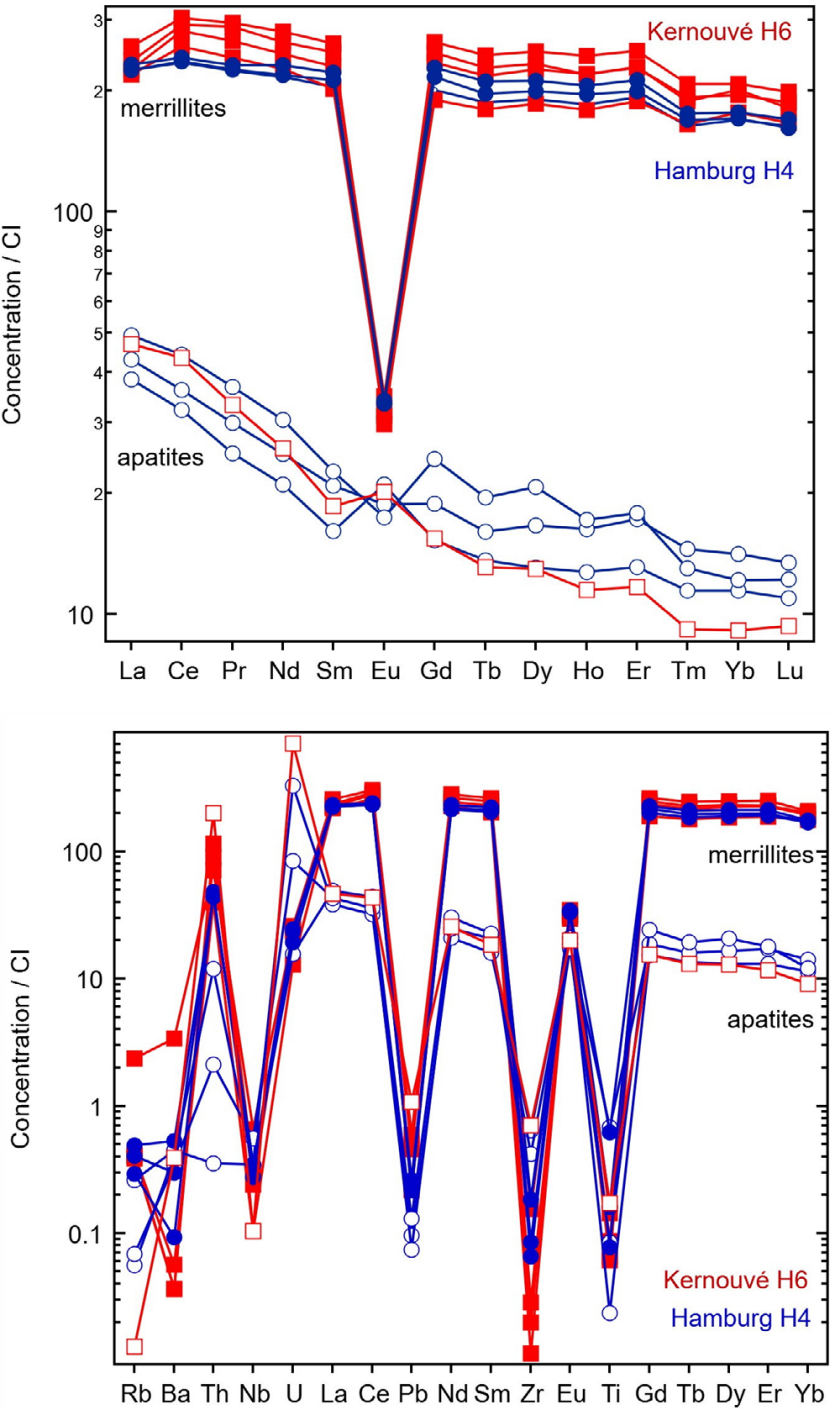
Trace elemental geochemistry (normalized to CI chondrites) by LA‐qICPMS analysis of apatite and merrillite grains of Hamburg (H4; blue round symbols, filled for merrillites, open for apatites) and Kernouvé (H6; red square symbols, filled for merrillites, open for apatites) for comparison. (Color figure can be viewed at wileyonlinelibrary.com.)

Before obtaining age data, we determined the concentrations of U, Pb, and other trace elements in phosphates. Phosphates are secondary phases formed by thermal metamorphism and, due to their relatively high U contents, can be used to obtain absolute radiometric ages of thermally metamorphosed ordinary chondrites (e.g., Wadhwa 2014). Apatite and merrillite in Hamburg H4 (Fig. 10) have trace element characteristics similar to corresponding mineral phases in Kernouvé H6 (Fig. 11), and previous reports for rare earth elements in chondritic merrillite found in the literature (e.g., Ward et al. 2017). Apatite is notably depleted in trace elements compared to merrillite except for U and Pb, which are highly variable and sometimes found in higher abundance in apatite grains (Tables S2 and S3 in supporting information). Merrillite grains have strong negative anomalies in Eu (Eu/Eu* = 0.15–0.17) compared to apatite (Eu/Eu* = 0.75–1.34) in Hamburg, similar to Kernouvé (merrillite Eu/Eu* = 0.12–0.16 and apatite Eu/Eu* = 1.18). In Hamburg, apatite has low Th/U (0.1–0.3) compared to merrillite (3.7–8.3), similar to Kernouvé, which has Th/U 0.6–1.0 in apatite and 8.0–16.2 in merrillite (Tables S1 and S2). Hamburg phosphates are overall slightly more depleted in U, Th, and Pb compared to those in Kernouvé. This is in accordance with the expectation that more intense thermal metamorphism leads to higher U concentrations in phosphates (Crozaz et al. 1989).

### U‐Pb Chronometry of Phosphates by LA‐ICPMS

Despite the small signal strengths of U and Pb in Hamburg (usually 10–20 counts per cycle), the data were plotted on a Tera–Wasserburg Reverse Concordia diagram. The U‐Pb data define reasonable ^207^Pb/^206^Pb ages and appear to contain negligible amounts of common Pb. The age determination does depend on a poorly constrained estimate of Pb isotope fractionation in the plasma during LA‐ICPMS analysis based on standard measurements. This fractionation is ~1%/AMU and favors heavier isotopes, resulting in measured ^207^Pb/^206^Pb ~1% too high. We find a U‐Pb Concordia age of 4595 ± 55 Ma for the eight phosphate grains measured in Hamburg (Fig. S5a in supporting information) and an average age of 4567 ± 43 Ma from the corresponding eight individual Pb‐Pb ages (Fig. S5b in supporting information). Individual precision was better on apatite spots owing to the higher U and Pb concentrations compared to merrillite, but the individual Pb‐Pb ages for both phosphates are identical within errors within our data set. We note that among the eight phosphate grains successfully analyzed by LA‐ICPMS, three merrillite analyses may be excluded from age calculations (Table S3). Merrillite H1 has a higher Pb/U (1.12) compared to others (0.89 ± 0.05, 1SD), which suggests potential mixing with a Pb‐rich phase, and a model Pb‐Pb age >4.64 Ga. Merrillites H2 and H5 have much less precise Pb isotopic measurements (1SD >10% on ^207^Pb/^206^Pb) which are also associated with model ^207^Pb/^206^Pb ages older than 4.64 Ga. When regressing the five remaining analyses together, we find a Pb‐Pb average age of 4549 ± 36 Ma (95% confidence level, MSWD = 0.59; Table S2). Because the U‐Pb data are tightly grouped (Fig. S5a) resulting in a poorly constrained fit, a “Concordia age” calculation could not be performed.

Kernouvé (polished section #USNM 2211 b) is another H fall with phosphates large enough (>30 µm) for LA‐ICPMS U‐Pb analyses. Importantly, the age of Kernouvé phosphates was precisely reported by the conventional TIMS Pb‐Pb method (Göpel et al. 1994) so that we could compare with our LA‐ICPMS data on Kernouvé and assess the accuracy of our measurements and age determination. Kernouvé has larger and less fractured phosphates, which allowed ablation with a larger 40 µm beam. The average age for six phosphates in Kernouvé is 4515 ± 26 Ma for the U‐Pb Concordia age (Fig. S6a in supporting information) and 4514 ± 16 Ma from six individual Pb‐Pb ages (Fig. S6b in supporting information). These ages are consistent with more precise ID‐TIMS results on phosphate separates of Kernouvé with an average Pb‐Pb age of 4521 ± 1 Ma recalculated using ^238^U/^235^U = 137.79 (*n* = 2; Göpel et al. 1994).

### U‐Pb Chronometry of Phosphates by SIMS

**Fig. 12:**
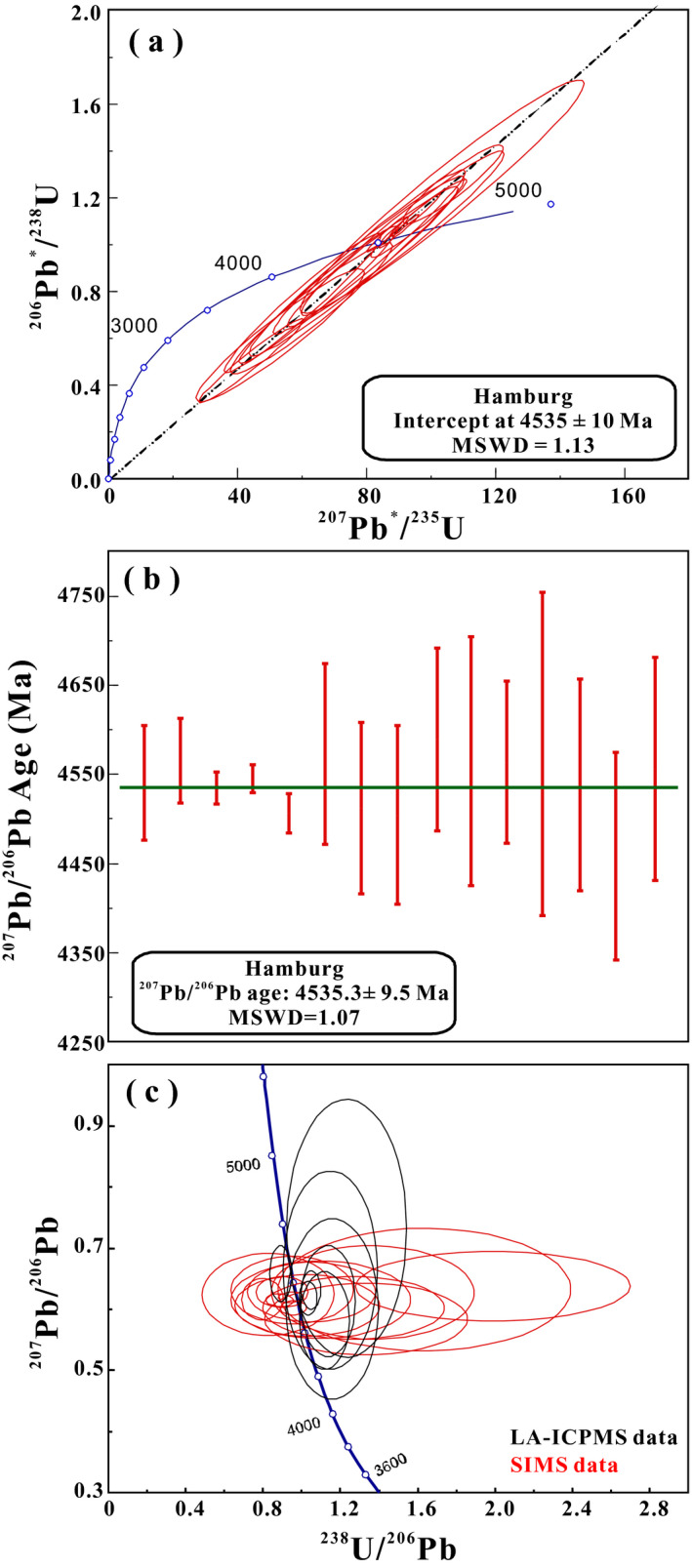
SIMS measurements with U‐Pb age in Wetherill concordia diagram (a) and Pb‐Pb age (b) of phosphates (merrillites and apatites) from Hamburg H4 chondrite. Panel (c) shows the comparison of the SIMS data with those of LA‐ICP‐MS data in a Tera–Wasserburg diagram. (Color figure can be viewed at wileyonlinelibrary.com.)

Besides LA‐ICPMS, we have used SIMS to determine U‐Pb and Pb‐Pb ages in phosphates of Hamburg. The SIMS U‐Pb data are presented in Table S4 and position of spots is shown in Fig. S7 in supporting information. The U‐Pb Concordia age of 15 spot SIMS analyses is 4535 ± 10 Ma (Fig. 12a). The corresponding Pb‐Pb average age is 4535.3 ± 9.5 Ma (95% confidence level, MSWD = 1.07; Fig. 12b) with precision on ^207^Pb/^206^Pb ratios ranging from 0.6% to 5.0% (Table S4).

Hamburg phosphates have an average Pb‐Pb age of 4549 ± 36 Ma (*n* = 5) by LA‐ICPMS analysis and 4535.3 ± 9.5 Ma (n = 14) by SIMS (Figs. 12 and S5); both are consistent within errors. Taken together, the weighted average Pb‐Pb age for selected phosphates from all SIMS and LA‐ICPMS data is 4541.6 ± 9.5 Ma (95% confidence level, MSWD = 3.6, *n* = 19). This formation age is intermediate between other H4 chondrite phosphate ages reported previously for Forest Vale (4560 ± 1 Ma; Göpel et al. 1994) and Sainte Marguerite (4562 ± 1 Ma; Bouvier et al. 2007) and Avanhandava (4516 ± 2 Ma; Blackburn et al. 2017). The age for Hamburg is most similar to metamorphic type 5 ordinary chondrite phosphates ranging from 4549 to 4554 Ma for Allegan, Nadiabondi, and Richardton (Göpel et al. 1994). Hamburg phosphate ages are older than H6 phosphate ages for Kernouvé (4514 ± 16 Ma in this study, 4521 ± 1 Ma in Göpel et al. 1994) and ALHA 78116 (4507 ± 2 Ma; Blackburn et al. 2017). The age of Hamburg phosphate falls within the period of crystallization of phosphates during thermal metamorphism following accretion of the H chondrite parent body (Göpel et al. 1994; Bouvier et al. 2007). Hamburg has an estimated S2 shock level. We find no evidence of disturbance by late events such as found in the phosphate U‐Pb ages of shocked L chondrites reported at ~4.4 Ga and ~0.67 Ga, respectively (e.g., Li and Hsu 2018a, 2018b; Wu and Hsu 2017; Yin et al. 2014).

Plotting SIMS and LA‐ICP‐MS data together on a reverse Tera–Wasserburg Concordia illustrates the two methods (Fig. 12c). SIMS U/Pb errors (*x*‐axis) are larger whereas LA‐ICPMS ^207^Pb/^206^Pb errors (*y*‐axis) are larger; both could be improved in future investigations. For SIMS measurements, the Pb^+^ ion yield is >25 cps/ppm/nA, while U^+^ is only 4 cps/ppm/nA. The uncertainty of the U/Pb ratio is dominated by the error in the U measurements. For a more precise measurement for this type of sample in the future, the U‐Pb dating procedure should perhaps use Pb/UO versus UO_2_/UO correlation to control elemental fractionation. The UO^+^ ion yield is around five to six times higher than U^+^. In this study, we used Pb/U versus UO_2_/U instead to calibrate against an apatite standard NW‐1. We anticipate this would improve the precision of the U/Pb measurements in the future. The main weakness of the LA‐ICPMS data is the limitation in accuracy of ^207^Pb/^206^Pb measurements because of uncertainty in the exact Pb fractionation in the plasma, something that could be controlled and determined by measuring a NIST standard during the run. For the SIMS ^207^Pb/^206^Pb analyses, the observed mass fractionation was only 0.36% to −0.24% (Stern et al. 2009). This is negligibly small compared to the actual measurement uncertainty of a few percent of this ratio (see Table S4). Therefore, the SIMS ^207^Pb/^206^Pb measured ratios are more correctly determined than for LA‐ICPMS.

### ^40^Ar/^39^Ar and U‐Th/He‐Chronometry

**Fig. 13:**
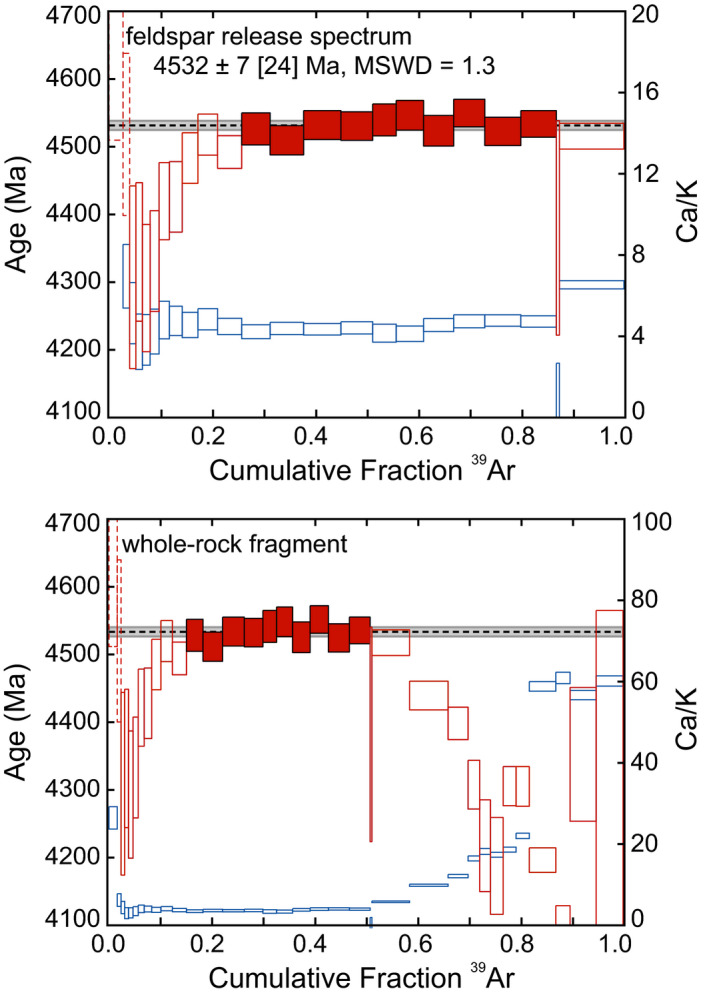
^40^Ar/^39^Ar age and Ca/K spectra obtained from a whole‐rock fragment. ^40^Ar/^39^Ar ages are shown in red and are plotted against the primary *y*‐axis. Ca/K spectra are shown in blue and are plotted against the secondary *y*‐axis. Each spectrum is plotted against the cumulative fraction of ^39^Ar released. Vertical dimensions of the boxes reflect the ±2σ analytical uncertainties. The horizontal dashed black lines and associated gray bands reflect the plateau age and associated 2 SE uncertainty, respectively. Filled boxes denote steps that were included in the plateau age. Dashed boxes at low extraction temperatures have appreciable terrestrial atmospheric contamination. The upper panel highlights the feldspar portion of the age spectrum, which comprises extractions that precede the abrupt increase in Ca/K associated with the onset of pyroxene degassing. The bottom panel shows the entire whole‐rock release spectrum. Pyroxene extractions appear to be significantly affected by ^39^Ar recoil, which results in sub‐plateau ages. (Color figure can be viewed at wileyonlinelibrary.com.)

^40^Ar/^39^Ar ages are less precise than U‐Pb ages, but they are useful for dating thermal events. The ^40^Ar/^39^Ar results obtained from the incremental degassing of a whole‐rock fragment of Hamburg are shown in Fig. 13 and Table S5 in supporting information. Discordant, sub‐plateau ages were obtained at low temperatures (low cumulative ^39^Ar release fractions), likely the result of one or more rather moderate thermal occurring events after 4.3 Ga. Likewise, at high temperatures (high cumulative ^39^Ar release fractions), sub‐plateau ages were obtained from pyroxene‐derived gas extractions with elevated Ca/K ratios (>10). Given the fine grain size of matrix feldspars and pyroxenes, high‐temperature discordance is likely attributable to recoil‐implanted ^39^Ar from potassium‐rich feldspar into potassium‐poor pyroxene. Intermediate temperature steps associated with the feldspar portion of release spectrum define a plateau age of 4532 ± 7 [24] Ma (2σ; MSWD = 1.3; the uncertainty in brackets includes the standard age, decay constant, and J‐value, which is related to the neutron fluence, uncertainties). The Hamburg plateau age is consistent with other ^40^Ar/^39^Ar ages obtained from H4 chondrites (e.g., Trieloff et al. 2003). Hamburg’s ages based on U‐Pb, Pb‐Pb, and ^40^Ar/^39^Ar all agree within uncertainties; this indicates that Hamburg did not experience a major thermal event since its formation.

We also determined the U‐Th/He ages of two aliquots of Hamburg. The apparent U‐Th/He ages of 3.0 ± 0.3 and 3.2 ± 0.3 Ga are consistent with other U‐Th/He ages obtained from H chondrites, which generally fall between 3.0 and 4.2 Ga (e.g., Wasson and Wang 1991). Helium is easily lost in comparison to Ar and Pb. These younger U‐Th/He ages likely reflect ^4^He loss during ejection from a near‐Earth asteroid and/or loss during ejection from the parent body. The temperatures and durations associated with He loss were insufficient to extensively reset the K‐Ar system, but likely explain the sub‐plateau ages observed at low extraction temperatures.

### Cosmogenic Nuclides

Nuclides produced by spallation reactions from galactic cosmic rays enable the determination of the CRE age, that is, the interplanetary residence time of a meteoroid. Here, we measured isotopes of the light noble gases He, Ne, and Ar and cosmogenic radionuclides ^10^Be and ^26^Al. He, Ne, and Ar isotopes were measured in two unirradiated, whole‐rock fragments weighing 4.07 and 6.15 mg. The data are given in Table S6 in supporting information. The smaller fragment appears to have been metal‐rich based on elevated ^38^Ar_cos_ and depressed ^21^Ne_cos_ relative to the larger fragment and discordant exposure ages calculated using the chemical composition of H4 chondrites. As such, the following discussion focuses on data obtained from the larger whole‐rock fragment, which yielded concordant ages. The cosmogenic ^22^Ne/^21^Ne ratio, which is an indicator for the shielding depth during cosmic ray irradiation, is 1.08 ± 0.02 (average of all gas released except the first and last extractions, which contain noncosmogenic Ne). This ratio is consistent with irradiation near the center of a meteoroid with a radius of 20–40 cm or at intermediate depths in a meteoroid with a radius between 50 and 100 cm (Leya and Masarik 2009).

**Fig. 14:**
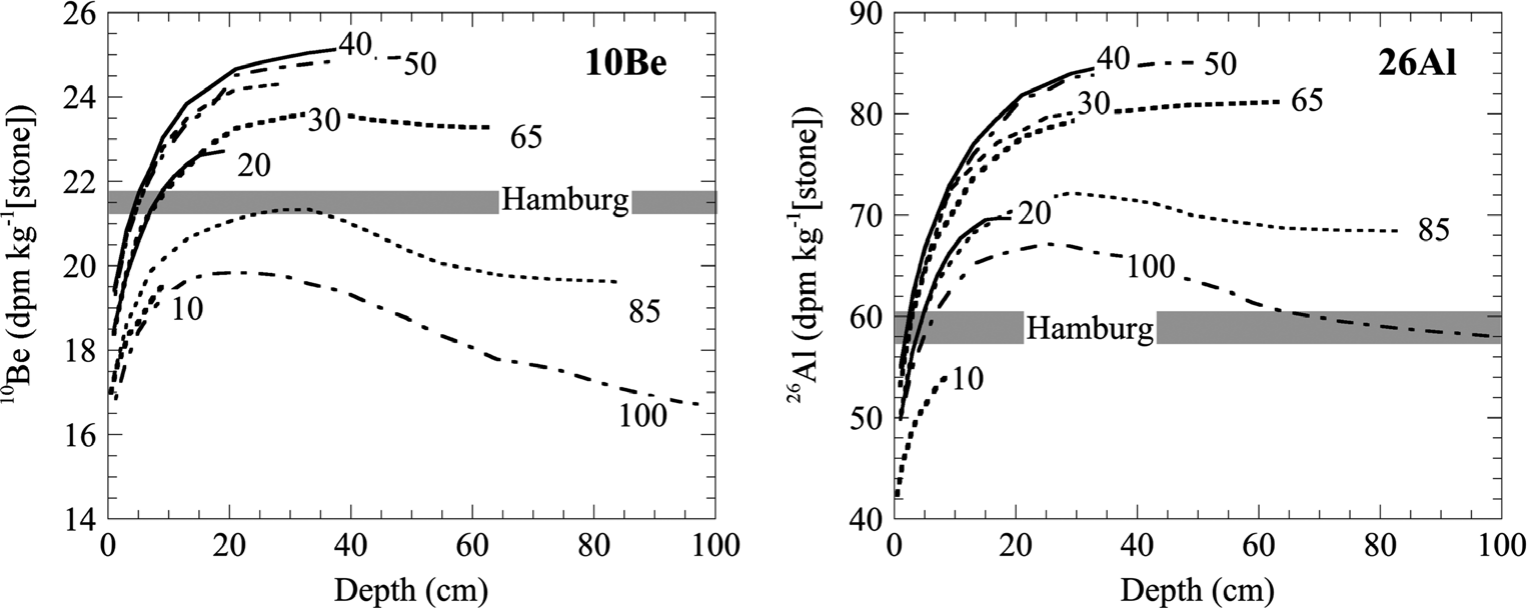
Comparison of measured ^10^Be and ^26^Al concentrations in the non‐magnetic (“stone”) fraction of Hamburg (H4), represented by the gray bar, with calculated ^10^Be and ^26^Al production rates in the stone fraction of H‐chondrites with radii of 10–100 cm (Leya and Masarik 2009).

The measured ^10^Be and ^26^Al concentrations of 21.5 ± 0.2 dpm kg^–1^ and 59.0 ± 1.2 dpm kg^–1^ in the nonmagnetic (“stone”) fraction of Hamburg (Fig. 14) are consistent with an irradiation duration of >10 Ma in the center of an object with a radius of ~15 cm, or near the surface (2–5 cm depth) of a larger object (20–65 cm radius). We can exclude the possibility that the sample was irradiated at the surface (<2 cm) of a larger object, as the measured ^26^Al concentration shows no evidence for solar cosmic ray‐produced ^26^Al. Together, these results suggest that the Hamburg meteoroid most likely had a relatively small radius of ~15 cm, corresponding to a preatmospheric mass of ~50 kg, although a larger size cannot be excluded without additional measurements.

The meteoroid size and depth of irradiation that best reproduce the measured ^21^Ne/^22^Ne ratio and minimize the relative standard deviation between the ^3^He, ^21^Ne, and ^38^Ar exposure ages is irradiation near the center of a meteoroid with a radius of 20 cm, although comparable fits can be obtained with irradiation near the surface of larger meteoroids. The ^3^He, ^21^Ne, and ^38^Ar CRE ages calculated for this irradiation scenario are 12.0 ± 1.2, 11.8 ± 1.2, and 11.6 ± 1.2 Ma, respectively. We conclude that the sample has a likely CRE age of ~ 12 Ma and originated from close to the center of a small meteoroid with a radius of 20–30 cm.

#### Clues for Hamburg’s Origin?

Hamburg’s CRE age of ~12 Ma indicates it is not associated with the collision event responsible for the 7 Ma peak in the H chondrite CRE distribution. Instead, the CRE age is similar to the ~15 Ma age of the H5 chondrite Pribram (Graf and Marti 1995), which arrived on a 10° inclined orbit with a semimajor axis *a* = 2.42 AU near the 3:1 resonance (Ceplecha 1961). Analysis of video observations of the Hamburg fall determined that the pre‐impact orbit had *i* = 0.6‐ ± 0.11° and *a* = 2.73 ± 0.05 AU (Brown et al. 2019). It is likely that Hamburg arrived to us also from the 3:1 resonance at *a* = 2.50 AU, but alternatively may have arrived to us via the 5:2 resonance at a = 2.82 AU. If Pribram and Hamburg originated from the same collision event, then orbital dynamics suggests it more likely originated from a source with an initial low ~1° inclined orbit, rather than a more highly inclined ~10° orbit (Jenniskens 2019). Inclinations from initial low‐inclined orbits tend to increase over time due to interactions with Earth (Jenniskens 2019). In contrast, the only observed H chondrite fall, where an orbit determination was made, with a CRE age of ~7 Ma belongs to a group of observed H chondrite falls with highly (24–32°) inclined orbits (Jenniskens 2019).

### Organic Chemistry

Characterizing the organic inventory of fresh, quickly recovered, and properly curated falls, such as Hamburg, is useful, as such meteorites typically are less contaminated with terrestrial organics. Contamination during terrestrial residence was observed in several meteorites (e.g., Zenobi et al. 1992). Every new meteorite has a specific profile of soluble carbon reflecting the history of organo‐mineral interactions and coevolution.

Here, we performed a comprehensive semiquantitative analysis of the soluble organic compounds in Hamburg. Great precautions were taken during the whole process from the sampling to the storage of the meteorite to avoid any terrestrial organic contamination. A fresh fragment was washed first and extracted while crushing to access the otherwise inaccessible organics of the matrices and in inclusions of the mineral phases.

**Fig. 15:**
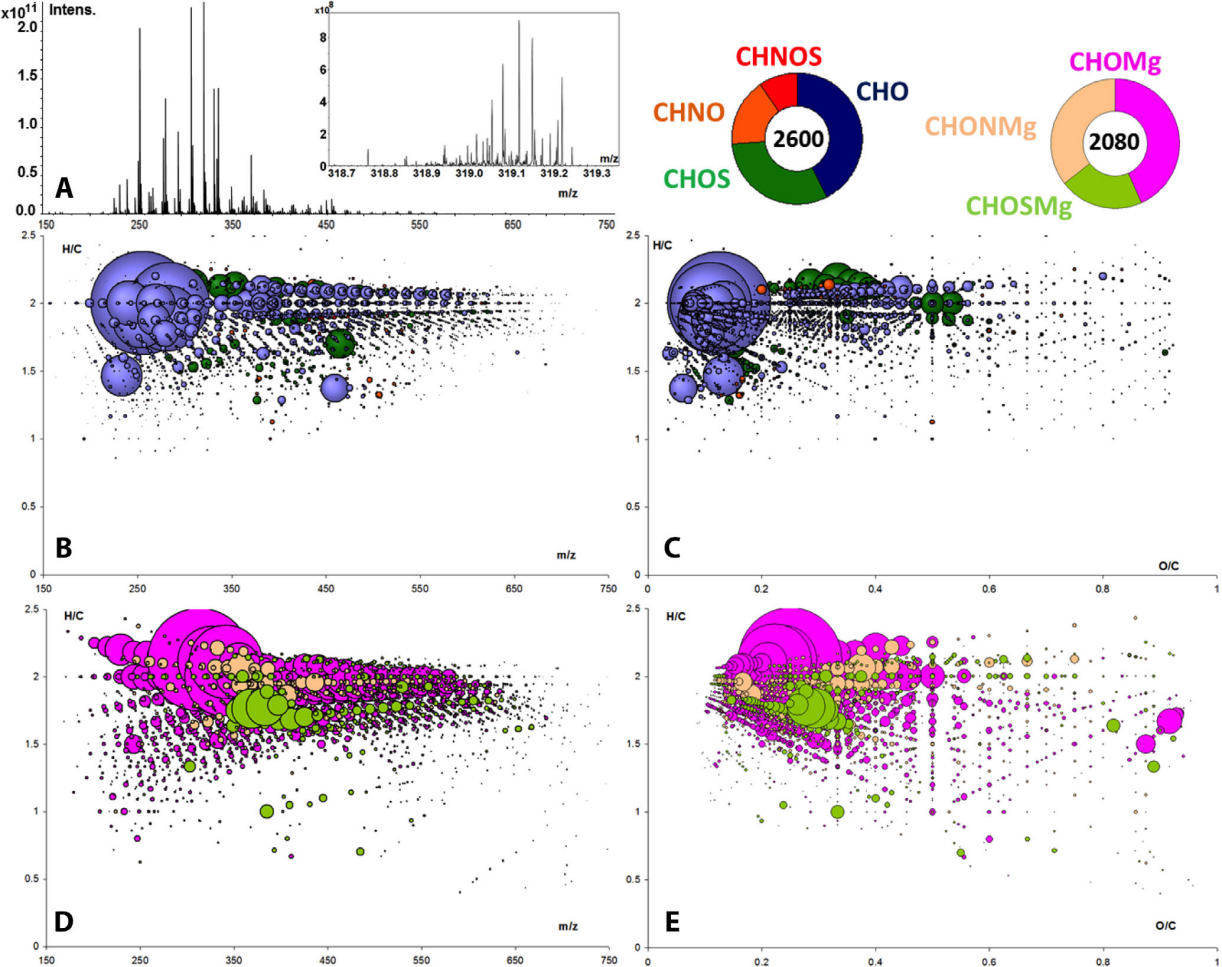
FTICR‐MS spectra of Hamburg methanol extract in electrospray negative ionization mode. A) FTIRC‐MS spectra in the whole mass range (150–750 amu) and detail on nominal mass 319 only, showing the most intensive signals and the signal diversity in the nominal masses, respectively. B/C) van Krevelen and mass resolved representation of the 2600 elementary compositions in the CHNOS space (the intensity of the signals in [A] has relative bubble sizes in the diagrams). D/E) 2080 magnesium containing compositions showing the relative highest intensity of CHOMg and high abundance also of nitrogen and sulfur containing organometallic compounds. (Color figure can be viewed at wileyonlinelibrary.com.)

The solvent soluble meteoritic organic matter of the Hamburg meteorite shows a high diversity of thousands of polar and sulfurized hydrocarbons (Fig. 15). The dynamic range in intensities of all the signals was 10^6^ with major signals corresponding to a homologous series of saturated fatty acids and sulfonated alkanes (>10^11^) and regular multiple signals (over 30) in each nominal mass (Fig. 15A). The exact mass analysis resulted in 2600 elementary compositions in the CHNOS elemental space with polar hydrocarbons being the most abundant followed by sulfur‐ and nitrogen‐containing compounds (Figs. 15B and 15C). By accounting for multiple isomers, we can confirm tens of thousands of structurally different complex organic molecules. The regular patterns in the van Krevelen graphs (e.g., Wu et al. 2004; Tziotis et al. 2011) correspond to the incremental changes in chemistry (mass) and abundance (intensity of the signals) with increasing molecular mass. The systematic mass increments of the signals in the van Krevelen graphs visualize chemical homologous series of the small molecules as witnesses of the history in chemical transformations (i.e., hydration, hydrogenation, hydroxylation, and methylation).

Our qualitative analysis shows that H chondrites also contain a high abundance of organic compounds (containing C, H, N, O, S) in accordance with our finding with other fresh LL and L chondrite falls (Popova et al. 2013; Jenniskens et al. 2014; Bischoff et al. 2019); a detailed description of the nomenclature of soluble organic matter in meteorites was recently given in the description of the highly shocked Renchen L5‐6 meteorite and compared to Stubenberg LL6 and Braunschweig L6 (Bischoff et al. 2019). The weakly shocked Hamburg meteorite also contains large amounts of organometallic compounds, which have previously been described as chemical markers for high temperature events (Ruf et al. 2017). In addition, we also found sulfur‐ and nitrogen‐containing organomagnesium compounds, further expanding this novel thermostable chemical class of compounds (Figs. 15D and 15E). The diversity of organic compounds is consistent with what is expected for the moderate thermal metamorphism and the very weak impact shock level that Hamburg experienced. From each new fresh meteorite analysis, we learn more about the close mineral and organic coevolution, and ongoing studies are trying to understand the process of formation of these novel chemical families and what mineral phases these may be associated with.

## Summary and Conclusions

About 1 kg of meteorite fragments was recovered in the Hamburg meteorite strewn field in Michigan, United States. Recovery was facilitated by the favorable terrain of snow‐covered frozen lakes. Radar reflections suggest a total surviving mass of only ~2 kg. Cosmogenic nuclide abundances suggest the meteorites fell from a small 40–60 cm sized meteoroid with a total mass of ~50 kg.

The petrology and mineral chemistry of this new meteorite are consistent with a very weakly shocked H4 chondrite with little to no terrestrial weathering effects. Triple oxygen isotopes and chromium isotopic compositions fall into the H4 chondrite field. Trace element characteristics of phosphates in Hamburg are similar to the Kernouvé H6 chondrite and other chondrites. Hamburg phosphates are overall slightly more depleted in U, Th, and Pb compared to those in Kernouvé.

The organic diversity is consistent with Hamburg’s moderate thermal metamorphism (H4) and very weak shock stage. Organic extract analysis shows that Hamburg contains 2600 elementary compounds in CHNOS space. Polar hydrocarbons are the most abundant, followed by sulfurized and N‐containing compounds. The distributions of these compounds suggest a series of chemical transformations (e.g., hydration, hydrogenation) occurring as parent body processes. Organometallic compounds are also present, with magnesium compounds being the most abundant.

CRE ages based on cosmogenic ^3^He, ^21^Ne, and ^38^Ar are 12.0 ± 1.2, 11.8 ± 1.2, and 11.6 ± 1.2 Ma, respectively, and roughly agree with each other. Hamburg did not originate from the collision event that produced the 7 Ma peak in the cosmic ray exposure distribution.

The last Ar‐releasing impact occurred 4532 ± 24 Ma ago, similar to what is observed for many other H chondrites. Our averaged Pb‐Pb ages of 4549 ± 36 Ma (LA‐ICPMS) and 4535.3 ± 9.5 Ma (SIMS) agree within error and reflect the metamorphic phosphate crystallization age after parent body formation. The Pb‐Pb ages (weighted average 4541.6 ± 9.5 Ma, *n* = 19) and ^40^Ar/^39^Ar age are identical within uncertainties and could represent the same event.

## Editorial Handling

Dr. Josep M. Trigo‐Rodríguez

## Supporting information


**Fig. S1.** a) Power law fit to the number of data from Table S1, showing the number of meteorites observed as a function of meteorite mass. b) Power law fit to the observed sum of meteorite mass seen in each radar sweep as a function of meteorite mass.
**Fig. S2.** Mass versus number of meteorites, plotted logarithmically for comparison with other meteorite falls.
**Fig. S3.** Backscattered electron image of typical feldspar (arrowed) in section ME 6108.3.
**Fig. S4.** a) Representative μCT images of Hamburg fragment 0.59 g taken at 10.65 μm isotropic voxel. This small fragment shows the rich FeNi (brightest white, outlined red in lower panel) and Fe‐Sulfide (slightly darker, outlined blue in lower panel) textures of this meteorite. Image intensity was adjusted to allow for visual differentiation of the FeNi and Fe‐Sulfide inclusions. b) Animation of μCT data.
**Fig. S5.** a) Concordia diagram from Hamburg U‐Pb LA‐ICPMS data. b) Pb‐Pb LA‐ICPMS data from Hamburg.
**Fig. S6.** U‐Pb and Pb‐Pb data from Kernouvé for comparison to Hamburg. a) Concordia diagram from Kernouvé U‐Pb LA‐ICPMS data. b) Pb‐Pb LA‐ICPMS data from Kernouvé.
**Fig. S7.** SIMS analyses spots of the phosphate minerals in Hamburg L4 chondrite with their petrographic context.

**Table S1**. Estimated masses and number of meteorites derived from radar data.
**Table S2**. Geochemical analyses with LA‐ICPMS of Hamburg ME 6108.3 and Kernouvé USNM 2211.
**Table S3.** U‐Pb geochronological analyses with LA‐ICPMS of Hamburg ME 6108.3 and Kernouvé USNM.
**Table S4.** SIMS U‐Pb isotopic data of phosphate from Hamburg (Specimen MSU‐Abrams 2018‐001).
**Table S5.** Complete ^40^Ar/^39^Ar incremental heating results of ME 6108.6.
**Table S6.** Helium, neon, and argon isotopic data of ME 6108.6.
