# Supplementary material for: The fall, recovery, classification, and initial characterization of the Hamburg, Michigan H4 chondrite
Source: Meteorit Planet Sci. 2020 Oct 27;55(11):2341–59. doi: 10.1111/maps.13584 (PMC7820957; doi:10.1111/maps.13584)
Supplement: Supplementary file 1 — Fig. S1. a) Power law fit to the number of data from Table S1, showing the number of meteorites observed as a function of meteorite mass. b) Power law fit to the observed sum of meteorite mass seen in each radar sweep as a function of meteorite mass. Fig. S2. Mass versus number of meteorites, plotted logarithmically for comparison with other meteorite falls. Fig. S3. Backscattered electron image of typical feldspar (arrowed) in section ME 6108.3. Fig. S4. a) Representative μCT images of Hamburg fragment 0.59 g taken at 10.65 μm isotropic voxel. This small fragment shows the rich FeNi (brightest white, outlined red in lower panel) and Fe‐Sulfide (slightly darker, outlined blue in lower panel) textures of this meteorite. Image intensity was adjusted to allow for visual differentiation of the FeNi and Fe‐Sulfide inclusions. b) Animation of μCT data. Fig. S5. a) Concordia diagram from Hamburg U‐Pb LA‐ICPMS data. b) Pb‐Pb LA‐ICPMS data from Hamburg. Fig. S6. U‐Pb and Pb‐Pb data from Kernouvé for comparison to Hamburg. a) Concordia diagram from Kernouvé U‐Pb LA‐ICPMS data. b) Pb‐Pb LA‐ICPMS data from Kernouvé. Fig. S7. SIMS analyses spots of the phosphate minerals in Hamburg L4 chondrite with their petrographic context. [file MAPS-55-2341-s001.docx]

Supporting Information

**The Fall, Recovery, Classification and Initial Characterization of the Hamburg, Michigan L4 Chondrite**

Philipp R. Heck^1,2^*, Jennika Greer^1,2^, Joseph S. Boesenberg^3^, Audrey Bouvier^4,5^, Marc W. Caffee^6^, William S. Cassata^7^, Catherine Corrigan^8^, Andrew M. Davis^1,2,9^, Donald W. Davis^10^, Marc Fries^11^, Mike Hankey^12^, Peter Jenniskens^13,14^, Philippe Schmitt-Kopplin^15^, Shannon Sheu^2^, Reto Trappitsch^7^, Michael Velbel^16,8^, Brandon Weller^17^, Kees Welten^18^, Qing-Zhu Yin^19^, Matthew E. Sanborn^19^, Karen Ziegler^20^, Douglas Rowland^21^, Kenneth L. Verosub^19^, Qin Zhou^22^, Yu Liu^23^, Guoqiang Tang^23^, Qiuli Li^23^ , Xianhua Li^23^, and Zoltan Zajacz^10^.

^1^Robert A. Pritzker Center for Meteoritics and Polar Studies, The Field Museum of Natural History, 1400 South Lake Shore Drive, Chicago, IL, 60605, USA.^*^corresponding author: prheck@fieldmuseum.org

^2^Chicago Center for Cosmochemistry and Department of the Geophysical Sciences, The University of Chicago, 5734 South Ellis Avenue, Chicago, IL 60637-1433, USA.

^3^Department of Earth, Environmental and Planetary Sciences, Brown University, 324 Brook Street, Box 1846, Providence, RI 02912, USA.

^4^Bayerisches Geoinstitut, Universität Bayreuth, Universitätsstraße 30, 95447 Bayreuth, Germany.

^5^Department of Earth Sciences, University of Western Ontario, BGS 1026, 1151 Richmond Street, London, ON, N6A 5B7, Canada.

^6^Department of Physics and Astronomy, Purdue University, West Lafayette, IN 47906, USA.

Department of Earth, Atmospheric, and Planetary Sciences, Purdue University, IN 47906, USA.

^7^Nuclear and Chemical Sciences Division, Lawrence Livermore National Laboratory, 7000 East Avenue (L-235), Livermore, CA 94550, USA.

^8^Department of Mineral Sciences, National Museum of Natural History, Smithsonian Institution, 10th St and Constitution Ave, NW, Washington, DC, USA.

^9^Enrico Fermi Institute, 5734 South Ellis Avenue, Chicago, IL 60637-1433, USA.

^10^Department of Earth Sciences University of Toronto, 22 Russell St, Toronto

Ontario, M5S 3B1, Canada.

^11^Astromaterials Research and Exploration Science Division, NASA Johnson Space Center, Mail Code XI2, Building 31, USA.

^12^American Meteor Society, 54 Westview Crescent, Geneseo NY 14454, USA.

^13^SETI Institute, 189 Bernardo Avenue, Mountain View, CA 94043, USA.

^14^NASA Ames Research Center, Moffett Field, CA 94035, USA.

^15^Helmholtz Zentrum München, Deutsches Forschungszentrum für Gesundheit und Umwelt (GmbH), Ingolstädter Landstr. 1, 85764 Neuherberg, Germany.

^16^Department of Earth and Environmental Sciences, 288 Farm Lane, 207 Natural Sciences Building, Michigan State University, East Lansing, MI 48824, USA.

^17^Albany Medical College, 43 New Scotland Ave, Albany, NY 12208, USA.

^18^Space Sciences Laboratory, 7 Gauss Way, University of California, Berkeley, CA 94720-7450, USA.

^19^Department of Earth and Planetary Sciences, University of California at Davis, One Shields Avenue, Davis, CA 95616, USA.

^20^Institute of Meteoritics, University of New Mexico, 221 Yale Blvd NE, 313 Northrop Hall, Albuquerque, NM 87131, USA.

^21^Center for Molecular and Genomic Imaging, University of California at Davis, Davis, CA 95616, USA.

^22^National Astronomical Observatories, Chinese Academy of Sciences, Beijing 100012, China.

^23^State Key Laboratory of Lithospheric Evolution, Institute of Geology and Geophysics, Chinese Academy of Sciences, Beijing 100029, China.

Supporting Information: Methods

Supplementary Figures: S1-S7 (this file)

Supplementary Tables: S1-S6 (In separate XLSX file)

**METHODS**

**Weather radar**

Data from two weather radars, the KDTX WSR-88D radar in Detroit, MI and the TDTW Terminal Doppler Weather Radar (TDWR) that serves the Detroit Metropolitan Airport were used to acquire reflection data from the meteorite fall. The KDTX radar is operated as part of the National Oceanographic and Atmospheric Administration’s (NOAA) NEXRAD nationwide radar system, and the TDTW radar is operated by the Federal Aviation Administration (FAA). Data from both radars are available through the NOAA NEXRAD online database. Meteorite masses were calculated using the Jörmungandr dark flight model devised by Fries and Fries (2010a, 2010b, 2010c) and Fries et al. (2011, 2012, 2013, 2016) by finding the size of a sphere of H chondrite density (3.4 g.cm^3^; Britt and Consolmagno 2003; Wilkison et al. 2003) that traverses the distance between the terminus altitude and the measured radar signature, in the difference in time between them. Measurements of wind direction and windspeed are used in the model, and taken from a NOAA radiosonde (e.g. “weather balloon”) launched from White Lake, MI at 0000 UTC on 17 January 2018. The total mass of meteorite falls can be estimated by comparing the calculated radar reflectivity of a given meteorite with the measured total reflectivity per image pixel, in a similar manner to that employed to estimate the number of migrating birds seen in weather radar data (Gauthreaux et al. 1998; Schmaljohann et al. 2008). Each radar sweep contains a total amount of radar energy reflected back to the radar from falling meteorites. With the estimate of meteorite mass for each sweep, the expected radar energy reflected from a single meteorite can be calculated and from that a total number of meteorites observed in the radar sweep can be estimated. For this calculation, only the three data points from the NEXRAD radar were used (Table S1) because a reliable method for directly comparing data between NEXRAD and TDWR radars has not yet been demonstrated. Meteorite mass can be expressed several ways, to include meteorite number distribution and total observed mass distribution (Fig. S1), and the mass distribution provides a direct comparison against other falls when plotted on a logarithmic scale for clarity (Fig. S2).

**Magnetic measurements**

Magnetic measurements were carried out on a 0.59 g Hamburg sample (MSU-Abrams Planetarium specimen 2018-001) at the University of California at Davis. Magnetic susceptibility was measured using a Bartington MS-2 Magnetic Susceptibility Bridge with a MS-2B dual-frequency sensor at UC Davis. The magnetic remanence of the Hamburg sample was then measured using a 2G Enterprises Model 755 automated cryogenic magnetometer.

**Computed micro-tomography**

A thin slab of the Hamburg meteorite (MSU-Abrams Planetarium specimen 2018-001) was imaged at the Center for Molecular and Genomic Imaging (UC Davis) with high-resolution X-ray CT. The sample was wrapped in tissue paper and secured in place using a custom plastic holder. X-ray tomographic images were obtained on a MicroXCT-200 specimen CT scanner (Carl Zeiss X-ray Microscopy). The CT scanner has a variable X-ray source capable of a voltage range of 20-90 kV with 1-8 W power. The sample was mounted on the scanner’s sample stage, which has sub-µm level of position adjustments. Scan parameters were adjusted based on the manufacturers recommended guidelines. First the source and detector distances were adjusted based on sample size and the optimal field of view for the given region of interest. Once the source and detector settings were established, the optimal X-ray filtration was determined by selecting among one of 12 proprietary filters. LE5 was the filtration selected. Following this procedure, the optimal voltage and power settings were determined for optimal contrast (75 kV and 105 µA). 1600 projections were obtained over a 360-degree rotation. The camera pixels were binned by 2 to increase the signal to noise in the image. The “0.4×” detector was used with source-to-sample and detector-to-sample distances of 25 mm and 134 mm, respectively. Images were reconstructed and visualized in Amira Software (Thermo Scientific).

**Sample preparation for microanalysis**

A fragment was removed, using a diamond wafering blade in a Buehler Isomet low-speed saw lubricated with water-free high-purity isopropanol (Optima™), from the complete 24.1 g Strawberry Lake individual of the Hamburg meteorite FMNH ME 6108.1 (Fig. 4). A fragment from this individual (FMNH ME 6108.3) was embedded in Buehler EpoFix epoxy and polished with Allied diamond lapping film and Optima™ isopropanol. The sample was then imaged in reflected light with optical microscopy at the Field Museum’s Robert A. Pritzker Center for Meteoritics and Polar Studies. Another section was prepared conventionally with water from MSU-Abrams Planetarium specimen 2018-001. A single thin section was prepared from a 0.204 g, 10 × 5 × 3 mm slice of MSU-Abrams Planetarium specimen 2018-001, a 17 g individual recovered later on 18 January 2019 from the ice on Strawberry Lake. Optical reflected-light images of this second studied specimen were acquired at MSU’s Center for Advanced Microscopy.

**Electron microscopy**

After carbon coating, the polished section was imaged and analyzed with a scanning electron microscope (SEM). To study the polished section (ME6108.3) from the type specimen we used the Field Museum’s Zeiss Evo 60 W-filament SEM equipped with an Oxford Instruments X-Max 50 silicon drift detector, and the University of Chicago’s field emission TESCAN LYRA3 FIB-FE-SEM equipped with two Oxford Instruments X-Max-80 silicon drift detectors. Both SEMs were run at 15 kV acceleration voltage. The Evo 60 was run at ~2.5 nA and the LYRA3 at ~2 nA. We used a set of mineral standards to calibrate the energy dispersive spectroscopy (EDS) system, calibrated and monitored stability of the e-beam current by using bracketed analyses of a Co standard in between 10-20 sample spots. Elemental data with totals <98% or >102% were rejected. We used Oxford Instruments AZtec software to prepare maps, and to obtain quantitative calibrated and corrected EDS data. We prepared RGB EDS maps using Adobe Photoshop™️. Modal abundances of minerals and morphometrics of textural features were obtained using the Fiji image processing software (Schindelin et al. 2012).

Backscattered electron images (BEI) of the Michigan State University Abrams Planetarium specimen 2018-001 and thin-section 2018-001a-TS were acquired on the FEI Nova NanoSEM 600 in the Department of Mineral Sciences (DMS) at the Smithsonian Institution’s (SI) National Museum of Natural History (NMNH).

**Raman spectroscopy**

To classify minerals in the type specimen, a WITec Alpha300 confocal Raman microscope system at the Robert A. Pritzker Center was used, with a 532 nm diode laser at a laser power of ~5 mW was used. The instrument conditions were monitored multiple times in each session by analyzing a standard Si wafer and recording the position of the major Si peak. Wavenumber shifts of the reference Si peak were typically less than one wavenumber (cm^–1^).

**Electron microprobe**

Eleven chondrules of the MSU specimen were selected for SEM imaging, and the ten largest for electron probe microanalysis (EPMA) characterization. EPMA was conducted used the JEOL JXA-8530FPlus HyperProbe Electron Probe Microanalyzer at the Smithsonian (NMNH, DMS). Chondrule silicates were analyzed for Si, Al, Fe, Mn, K, Ca, Ti, Mg, Na, and Cr. Standards were bytownite, Springwater olivine, Kakanui hornblende, and manganite. The microprobe was operated at 15 kV, 20 nA, using spot mode; transects of closely spaced spots were acquired across entire olivine and pyroxene crystals to characterize compositional zoning.

Phosphate grains in the type specimen section were first mapped by BSE and EDS on a thick polished mount at the University of Chicago. Major element, F, and Cl abundances were used to select apatite and merrillites for trace element analyses and U-Pb chronometry by LA-ICPMS at the University of Toronto.

Quantitative analysis on olivine, pyroxene, chromite and metal was performed on a thick section and a few chips of the Weller specimen of Hamburg utilizing the Brown University Cameca SX-100 electron microprobe. Operating conditions of the instrument consisted of 15 kV voltage, 20 nA current in spot mode. Counting times were 30 seconds for on-peak, and 15 seconds for backgrounds, for all elements. The data were calculated using the PAP correction procedures (Pouchou and Pichoir 1991). One-sigma standard deviations are <1% for major elements and 3–8% for minor elements. Standards used for the olivine and pyroxene analysis include Wakefield, Quebec diopside (Si, Ca); Kakanui pyrope NMNH 143968 (Al); synthetic Fo97 forsterite, University of Rhode Island (Mg); Rockport, Massachusetts fayalite, NMNH 85276 (Fe); Amelia albite, Purdue University (Na); rhodonite, AMNH 104738 (Mn); synthetic rutile, Brown University, (Ti); synthetic MgCr_2_O_4_ (Cr) and nickel metal, SPI 02751-AB-44 Metals Standards Serial AF (Ni). Standards for chromite included the same group as above except for synthetic MgAl_2_O_4_ (Mg, Al). For analysis of metal, Fe, Cr, Mn, Ni and Co standards all came from the SPI 02751-AB-44 Metals Standards Serial AF block. Phosphorus and sulfur standards consisted of synthetic berlinite (P) and Peru pyrite (S). Na in pyroxene was analyzed using a loss routine to account for any volatilization/migration of the element under the beam. Lunar Crater augite (NMNH 164905) and Tiebaghi Mine, New Caledonia chromite (NMNH 117075) were used as reference standards for olivine and pyroxene, and chromite analyses, respectively.

**Oxygen isotope analyses**

Oxygen triple isotope analyses of several subsamples of the meteorite (MSU-Abrams Planetarium specimen 2018-001) were performed by laser fluorination at the University of New Mexico (Sharp 1990). The bulk fragments were pretreated by acid-washing with weak HCl and subsequent rinsing in distilled water (for removal of possible terrestrial weathering products). Samples were prefluorinated (BrF_5_) in a vacuum chamber to clean the stainless steel system and to react residual traces of water or air in the fluorination chamber. Molecular oxygen was released from the samples by laser-assisted fluorination (50W far-infrared CO_2_ laser) in a BrF_5_-atmosphere, producing molecular O_2_ and solid fluorides. Excess BrF_5_ was then removed from the produced O_2_ by reaction with hot NaCl. The oxygen was purified by freezing onto a 13 Å molecular sieve at –196 °C, followed by elution of the O_2_ from the first sieve at ~300 °C into a He-stream that carries the oxygen through a CG column (separation of O_2_ and NF_3_, a possible interference with the ^17^O measurement) to a second 13 Å molecular sieve at **−**196°C. After removal of the He, the O_2_ is released directly into a dual inlet isotope ratio mass spectrometer (Thermo Finnigan MAT 253). The oxygen isotope ratios were calibrated against the isotopic composition of San Carlos olivine. Each sample analysis consisted of 20 cycles of sample-standard comparison. Olivine standards (~1–2 mg) were analyzed daily. Oxygen isotopic ratios were calculated using the following procedure: The δ^18^O values refer to the per-mil deviation in a sample (^18^O/^16^O) from SMOW, expressed as δ^18^O = [(^18^O/^16^O)_sample_/(^18^O/^16^O)SMOW–1]×10^3^. The delta values were converted to linearized values by calculating: δ^18^/^17^O’ = ln[(δ^18/17^O + 10^3^)/10^3^]×10^3^ to create straight-line mass-fractionation curves (Miller 2002). The Δ^17^O’ values were obtained from the linear δ–values by the following relationship: Δ^17^O’ = δ^17^O’–0.528×δ^18^O’; Δ^17^O’ values of zero define the terrestrial mass-fractionation line. Δ^17^O’ values lying on any line with a slope of ~0.52–0.53 are considered to be due to mass-dependent processes. Typical analytical precision of the laser-fluorination technique is better than ± 0.01‰ for Δ^17^O’.

**Chromium isotopic analyses**

Chromium isotopic measurements were made on a 19.68 mg subsample of Hamburg (MSU-Abrams Planetarium specimen 2018-001). The subsample was prepared by crushing a small, interior chip and digesting the powder in sealed PTFE Parr bombs with a 3:1 mixture of HF:HNO_3_ by placing in a 190°C oven for 96 hours. After digestion was complete, Cr was separated from the sample following the column separation procedure described by Yamakawa et al. (2009). The isolated Cr fraction was loaded onto outgassed W filaments (12 µg loaded evenly across four filaments). A terrestrial Cr standard NIST SRM 979 was loaded onto four filaments (3 µg load per filament) that were used to bracket the filaments containing the Cr separated from Hamburg.

Isotopic ratio measurements were made using a Thermo *Triton Plus* thermal ionization mass spectrometer at the University of California at Davis. A total of 1200 ratios (8 second integrations times) were measured for each filament. A gain calibration of the detectors was completed at the start of each filament and baselines measured every 25 ratios. Instrumental mass fractionation was corrected using an exponential mass fractionation law and a ^50^Cr/^52^Cr ratio of 0.051859 (Shields et al. 1966). The ^54^Cr/^52^Cr isotope ratio is expressed as parts per 10,000 deviation (ε-notation) from the NIST SRM 979 standard measured in the same analytical session.

**Trace element abundances in phosphates**

In-situ LA-ICPMS analyses of merrillite and apatite grains of polished section ME 6108.3 were carried out to obtain minor and trace element abundances (Table S2) and U-Pb radiometric ages (Table S3). Selected grains (based on size and compositions from EMPA) were laser ablated (LA) using an NWR193UC laser at 5 Hz and about 4 J/cm^2^ fluence with beam diameter of 20-40 µm, and analyzed on an Agilent 7900 quadrupole inductively-coupled plasma mass spectrometer (ICP-MS) at the University of Toronto. We first analyzed U-Th-Pb systematics of four merrillite and two apatites in the type specimen of Hamburg, and three merrillite and three apatites in Kernouvé H6 selected for comparison. During a second session, a suite of 42 major, minor and trace elements were measured on three apatite and three merrillite grains of Hamburg, and one apatite and four merrillite grains of Kernouvé. We monitored Ca contents on mass ^44^Ca and found them to be within error of EMPA Ca wt% contents of the corresponding grains and thus did not make any further corrections (Table S2).

**LA-ICPMS U-Pb dating of phosphates**

Precise (sub-Ma) age determination of chondrite phosphates have been previously obtained by wet chemistry on phosphate separates followed by Pb isotopic analysis by magnetic sector mass spectrometry using TIMS (Blackburn et al. 2017; Göpel et al. 1994) or multicollector-ICPMS methods (Bouvier et al. 2007). Such methods require mechanical extraction of small phosphate mixed grains from large crushed chondrite samples (typically 10–40 g of sample starting mass; (Blackburn et al. 2017; Bouvier et al. 2007; Göpel et al. 1994). Because of the limited amount of the Hamburg meteorite available for research (22 g at the Field Museum, ~1 kg in private collections), we used instead the polished mount made for the meteorite classification at the Field Museum (ME 6108.3) to analyze individual grains of phosphates for their geochemistry (by LA-ICPMS at Toronto) and U-Pb dating by LA-ICPMS (at Toronto) and SIMS (in Beijing) in-situ methods on the 0.59 gram slice (MSU-Abrams Planetarium specimen 2018-001). These methods are less destructive and provide location and textural relationships of the individual grains but compromise the precision to several million years or more for individual age dates. We report weighted averages of several individual grains of apatite and merrillite pooled together to improve precision on the age determination.

For Pb-Pb dating, data were collected on ^206^Pb (30 ms), ^207^Pb (30 ms), ^232^Th (10 ms) and ^238^U (30 ms). Following a 10 sec period of baseline accumulation the laser sampling beam was turned on and data were collected for 30 seconds followed by a 20 seconds washout period. About 150 measurement cycles per sample were produced and ablation pits are about 15–20 μm deep.

No corrections were made for common Pb, since the ^204^Pb peak was too small to be measured precisely and is subject to interference from ^204^Hg in the Ar gas carrier. Data were reduced using custom VBA software (UtilLAZ program) written by D. W. Davis. Because of the small size of the signals, time resolved profiles showing total counts, rather than counts/sec were calculated and ratios are based on total counts accumulated over the measurement, rather than the average of cycle ratios. This is a more statistically robust way of processing small signals and is explained in detail in Davis ( 1982).

Significant common Pb is usually present in terrestrial phosphates and has the effect of spreading the Pb-U composition along a mixing line between the concordant age (pure radiogenic Pb) and the ^207^Pb/^206^Pb composition of the common Pb component on a Tera-Wasserburg diagram (Tera and Wasserburg 1972a, 1972b). When plotted on a Wetherill Concordia diagram (Wetherill 1956), data affected by common Pb will scatter to the right along a mixing line whose slope is defined by the ^207^Pb/^206^Pb composition of the common Pb component. The Th/U ratio can be a useful petrogenetic indicator and was also measured, although it is only a rough estimate because the ratio is not constant in the standard (Table S3).

Two terrestrial apatite standards were analyzed with the samples. The MAD1 standard is a 485±1 Ma old gem quality apatite from Madagascar (Thomson et al. 2012). The 08Tanco-1 standard is from the Archean Tanco pegmatite and has been dated by isotope dilution thermal ionization mass spectrometry (ID-TIMS) at 2657±18 Ma (Camacho et al. 2012). The data reduction program was modified to take into account the fact that standards can only be constrained to a known mixing line. This requires an independent estimate of the ^207^Pb/^206^Pb fractionation in the plasma. This was taken to be 1.0±0.5% based on previous and subsequent results on standards of uniform composition such as zircon and NIST glass. Additionally, to assess the accuracy of our measurements, we also analyzed a polished sample of the H6 ordinary chondrite Kernouvé (polished section, Smithsonian Institution, #USNM 2211 b) which phosphates were previously analyzed for Pb-Pb dating by TIMS by Göpel et al. (1994).

**Secondary ion mass spectrometry**

A polished petrographic thick section from a 0.59 g Hamburg slab (MSU-Abrams Planetarium specimen 2018-001), after microCT scanning, was imaged with a Carl Zeiss SUPRA-55 field emission scanning electron microscope (FESEM) equipped with EDS at the National Astronomical Observatories (NAO), Chinese Academy of Sciences (CAS) in Beijing. Backscattered electron images and elemental Kα X-ray maps were acquired at an accelerating voltage of 15 kV. EDS elemental maps in P, Ca, Mg Kα were combined using Oxford Instrument’s AZtec software package in order to identify coarse phosphate grains including apatite [Ca_10_(PO_4_)_6_(OH,F,Cl)_2_] and merrillite Ca_18_Na_2_Mg_2_(PO_4_)_14_. This step was essential to select suitable analytical spots for the subsequent ion probe session to avoid micro-fractures, inclusions and other observed physical defects in the individual phosphate grains.

In situ isotopic analysis of U-Pb for phosphate grains was performed on the large radius magnetic sector multi-collector secondary ion mass spectrometer (SIMS), a Cameca IMS-1280HR at the Institute of Geology and Geophysics (IGG) at the Chinese Academy of Sciences (CAS) in Beijing. The detailed analytical procedure for U-Pb dating of terrestrial phosphate grains can be found in Li et al. (2012), which was further applied to meteoritic phosphates (Popova et al. 2013; Yin et al. 2014; Zhang et al. 2016; Zhou et al. 2018). Only a brief description is given here. The O_2_^–^ primary ion beam was accelerated at –13 kV, with an intensity ranging between 7 and 12 nA. The aperture illumination mode, Köhler illumination, was used with a 200 μm diameter aperture, resulting in an elliptical spot size of 20 x 30 μm^2^ on the target. Positive secondary ions were extracted with a 10 kV potential. A monocollector electron multiplier (EM) was used as the detection device to measure secondary ion beam intensities of ^204^Pb^+^, ^206^Pb^+^, ^207^Pb^+^, ^208^Pb^+^, ^232^Th^+^, ^238^U^+^, ^232^Th^16^O^+^_,_ ^238^U^16^O^+^_,_ ^238^U^16^O_2_^+^ and a matrix reference peak of ^40^Ca_2_^31^P^16^O_3_^+^ at a mass resolution of ~9,000 (defined at 50% height). The ^40^Ca_2_^31^P^16^O_3_^+^ signal was used as reference peak for tuning the secondary ions, energy, and mass adjustments. Pb/U ratios were calibrated with power law relationship between ^206^Pb*^+^/^238^U^+^ and ^238^U^16^O_2_^+^/^238^U^+^ relative to an apatite standard of NW-1 with an age of 1160 Ma that comes from the same complex at Prairie Lake as that of the apatite standard PRAP (Sano et al. 1999). U concentration is calibrated relative to the Durango apatite which has a U concentration of ~9 ppm (Trotter and Eggins 2006). The ^206^Pb/^238^U standard deviation measured in the standard was propagated to the unknowns. Each measurement consisted of 10 cycles, with a total analytical time of about 22 minutes. Uncertainties for individual analyses are reported as 1σ. The weighted average of ^206^Pb*/ ^238^U and Pb-Pb ages, quoted at the 95% confidence level, were calculated using ISOPLOT 3.0 (Ludwig 2003). In this study, correction of the common Pb was made by measuring the amount of ^204^Pb in the sample and the CDT Pb isotopic compositions (^206^Pb/^204^Pb = 9.307, ^207^Pb/^206^Pb = 1.09861; Tatsumoto et al., 1973). Data reduction used ^238^U/^235^U = 137.794±0.027 (Goldmann et al. 2015), the ^235^U decay constant of 9.8569x10^-10^/y (±0.017%) (Schoene et al. 2006), and ^238^U decay constant of 1.55125x10^-10^/y (±0.017%) (Jaffey et al. 1971).

**Noble gases**

Whole-rock fragments from the type specimen (ME 6108.6) were analyzed for ^40^Ar/^39^Ar, U-Th/He, and cosmic ray exposure ages in the Livermore Noble Gas Lab at Lawrence Livermore National Laboratory (LLNL). Detailed descriptions of the analytical procedures are provided in Cassata et al. (2018). Exposure ages were calculated from the element production rates given in Leya and Masarik (2009) using the chemical composition for H4 chondrites reported by Mason (1979) and Jarosewich (1990). All ^21^Ne was assumed to be cosmogenic. U-Th/He ages were calculated based on the U and Th concentrations reported by Shinotsuka et al. (1995) for the H5 chondrite Jilin. The ^40^Ar/^39^Ar plateau age was determined by including the maximum number of consecutive steps from the feldspathic portion of the age spectrum with a probability of fit >0.10. All ages are reported at 2σ. Cosmic ray exposure age uncertainties include an assumed 10% uncertainty on production rates. U-Th/He age uncertainties include an assumed 10% uncertainty to account for ambiguities in the U concentration. Complete analytical results are provided in the Supporting Information section available online.

**Cosmogenic radionuclides**

The concentrations of the cosmogenic radionuclides, ^10^Be (half-life = 1.36 Ma) and ^26^Al (half-life = 0.705 Ma), were obtained from a ~130 mg subsample of the type specimen (ME 6108.7). At UC Berkeley, the sample was gently crushed in an agate mortar and the powder was separated in non-magnetic ("stone") and magnetic (“metal”) parts using a magnet. An aliquot of 89.6 mg of the stone fraction was dissolved in concentrated HF/HNO_3_ along with a carrier solution containing approximately 3.0 mg of Be and 3.7 mg of Cl. After complete dissolution of the sample, a small aliquot of the solution was taken for chemical analysis by ICP-OES to determine the concentrations of the main target elements for radionuclide production. We added ~4.8 mg of Al to the remaining solution (to dilute the ^26^Al/Al ratio) and separated the radionuclides using routine ion exchange and acetyl-acetone extraction techniques. The Be and Al fractions were further purified and converted to the respective oxides, which were then mixed with Nb powder and loaded into stainless steel cathodes for measurement by Accelerator Mass Spectrometry (AMS) at Purdue University (Sharma et al. 2000). The measured ^10^Be/Be and ^26^Al/Al ratios were corrected for blank ratios of ~2.8×10^–14^ (^10^Be) and 1×10^–15^ (^26^Al) and normalized to well defined AMS ^10^Be and ^26^Al standards (Nishiizumi 2004; Nishiizumi et al. 2007).

**Organic chemistry**

Methanol extracts were prepared from an aliquot of the type specimen (ME 6108.4) and analyzed under the conditions as described by Schmitt-Kopplin et al., (2010) with negative ionization mode electrospray Fourier transform ion cyclotron resonance mass spectrometry (ESI(-)-FT-ICR-MS). About 50 mg of a fresh interior fragment of the type specimen of Hamburg was first washed with LC/MS grade methanol and immediately crushed in an agate mortar with 0.3 mL of methanol. After ultrasonicating for 1 minute, the sample was centrifuged for 3 min. All precautions were taken to avoid contaminations. A “blank” sample was analyzed before and after the meteorite analysis to confirm no organic interference from the analytical approach. Relative m/z errors were lower than 0.1 ppm across all the mass range of 150 < m/z < 1,000. FT-ICR-MS enabled an average mass resolution near 1,000,000 at nominal mass 200, 400,000 at mass 400 and 300,000 at mass 600. The same instrumental conditions were used as described earlier in Ruf et al. (2017) and Schmitt-Kopplin et al. (2010) to accumulate 3000 scans with 4 million data points. Data were calibrated and the conversion of the exact masses into elementary compositions is shown in more detail elsewhere (Tziotis et al. 2011).

**SUPPLEMENTARY FIGURES**
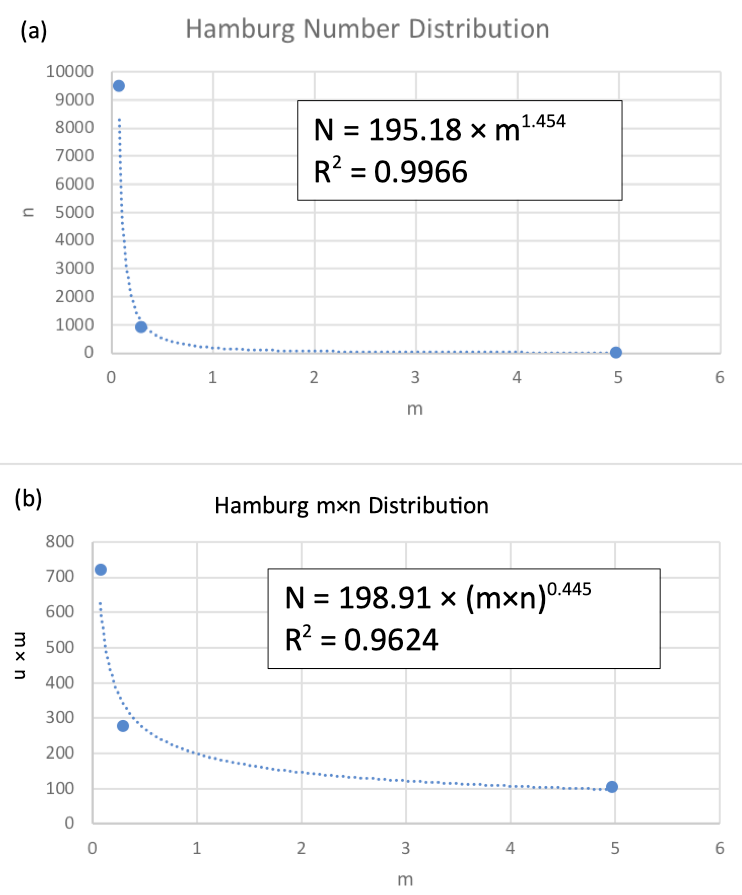


Figure S1: (a) Power-law fit to the number of data from Table S1, showing the number of meteorites observed as a function of meteorite mass. (b) Power-law fit to the observed sum of meteorite mass seen in each radar sweep as a function of meteorite mass. These data describe the function of fallen meteorite mass.


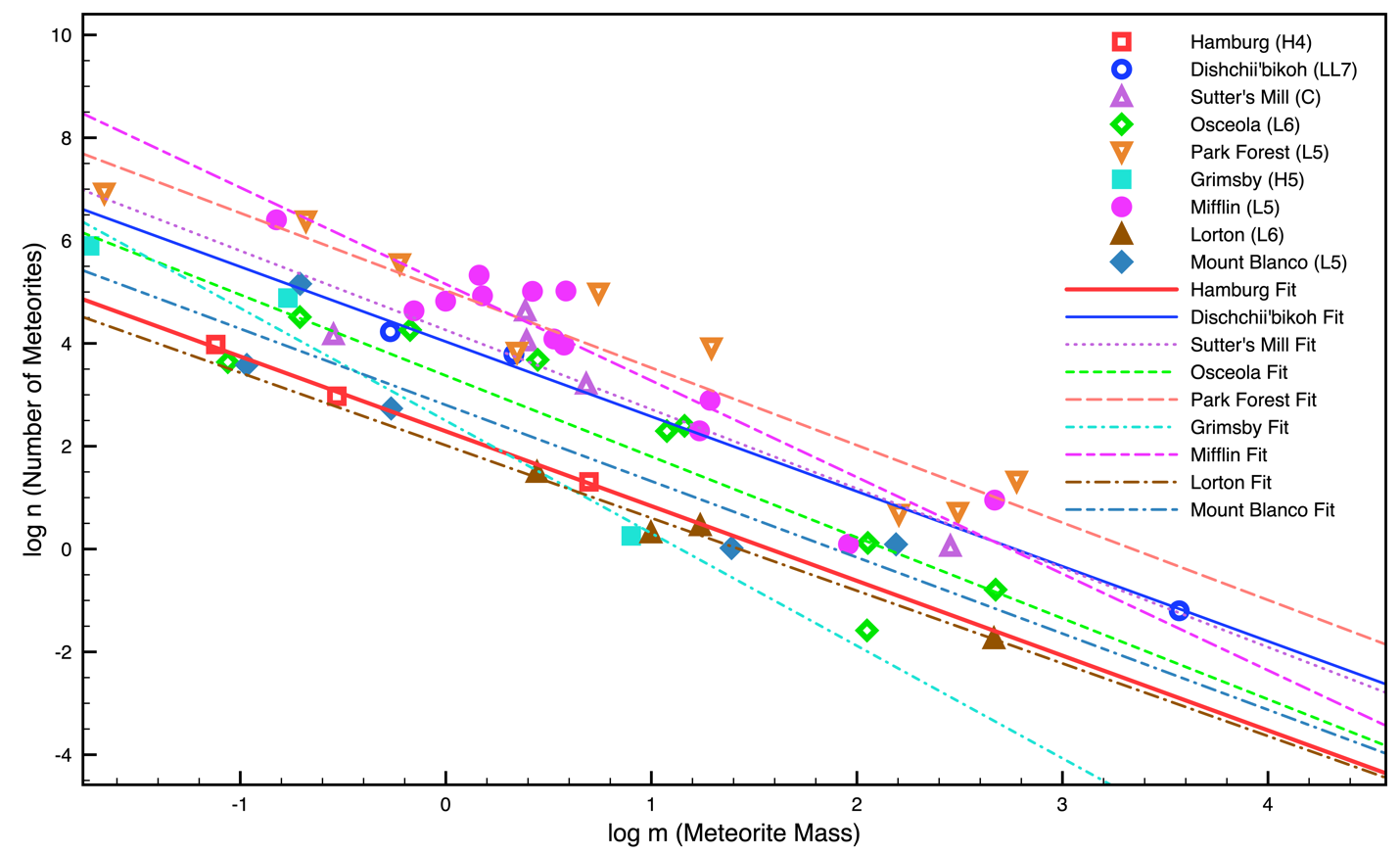


Figure S2. Mass versus number of meteorites, plotted logarithmically for comparison with other meteorite falls. In this graph, the higher up the line appears, the larger the total mass of the meteorite fall. The slope of the curve represents the relative abundance of larger-mass meteorites compared to the number of small meteorites that survived the fireball. Hamburg is shown in red. Only Lorton has a smaller total fall mass among the ten ordinary chondrite meteorite falls shown here, and the Hamburg line slope is comparable to most of the other meteorite falls. Note that Creston and Mifflin feature steeper slopes than the other falls, indicating fragmentation to preferentially produce small meteorite masses.


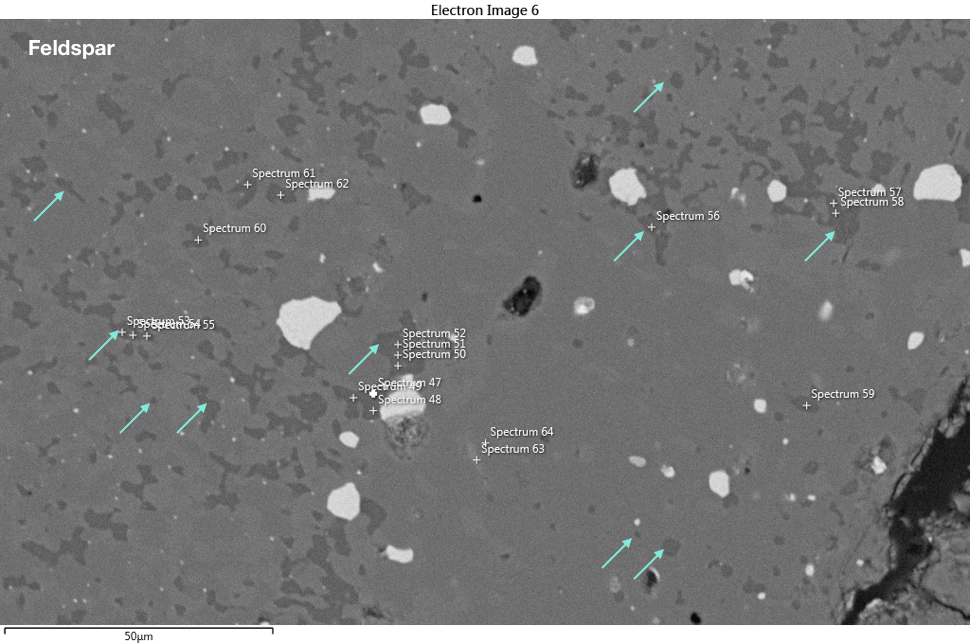
Figure S3. Backscattered electron image of typical feldspar (arrowed) in section ME 6108.3.

| (a)  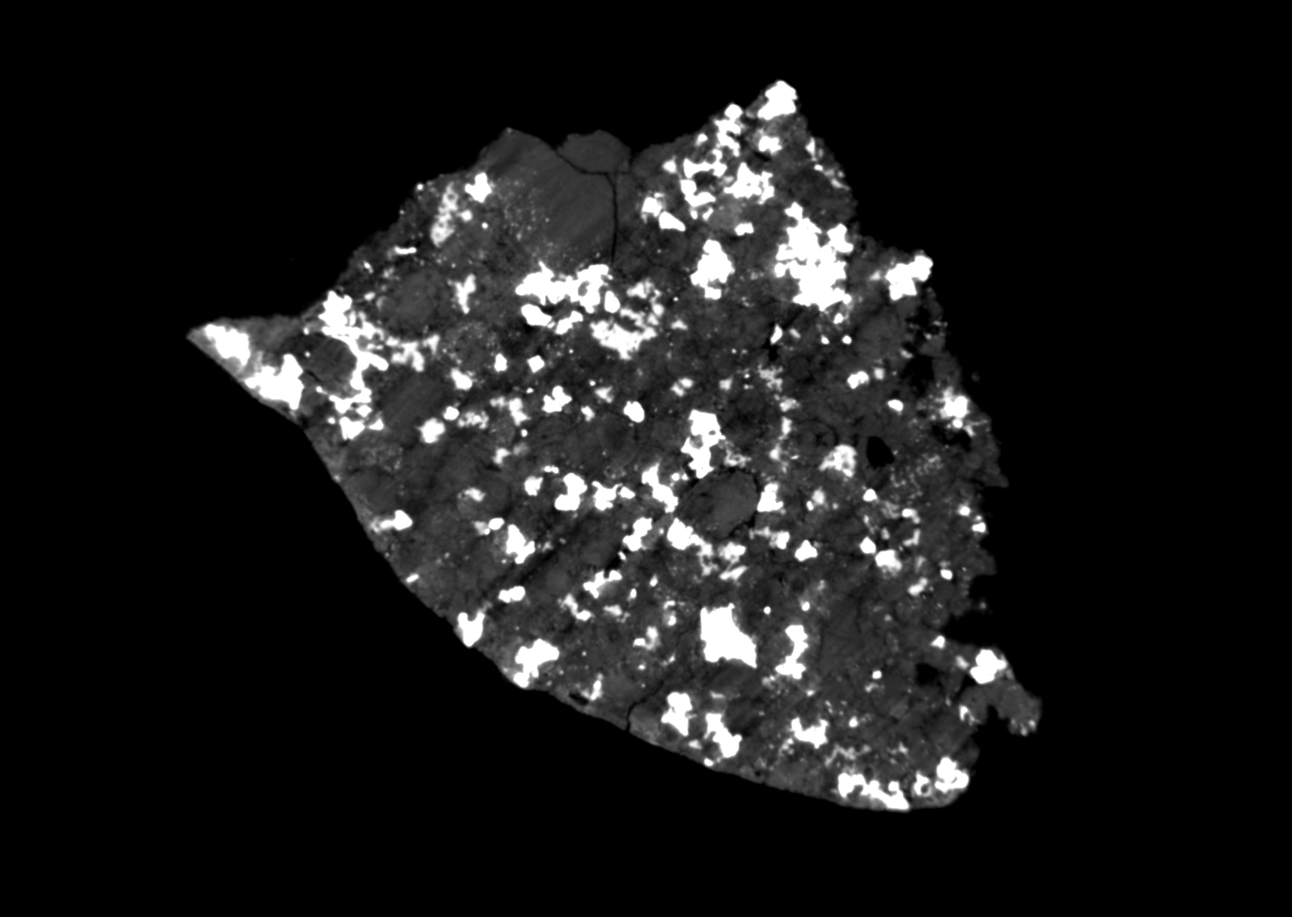  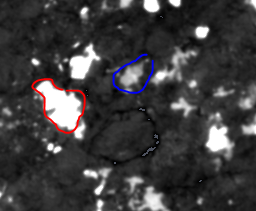 | (b)  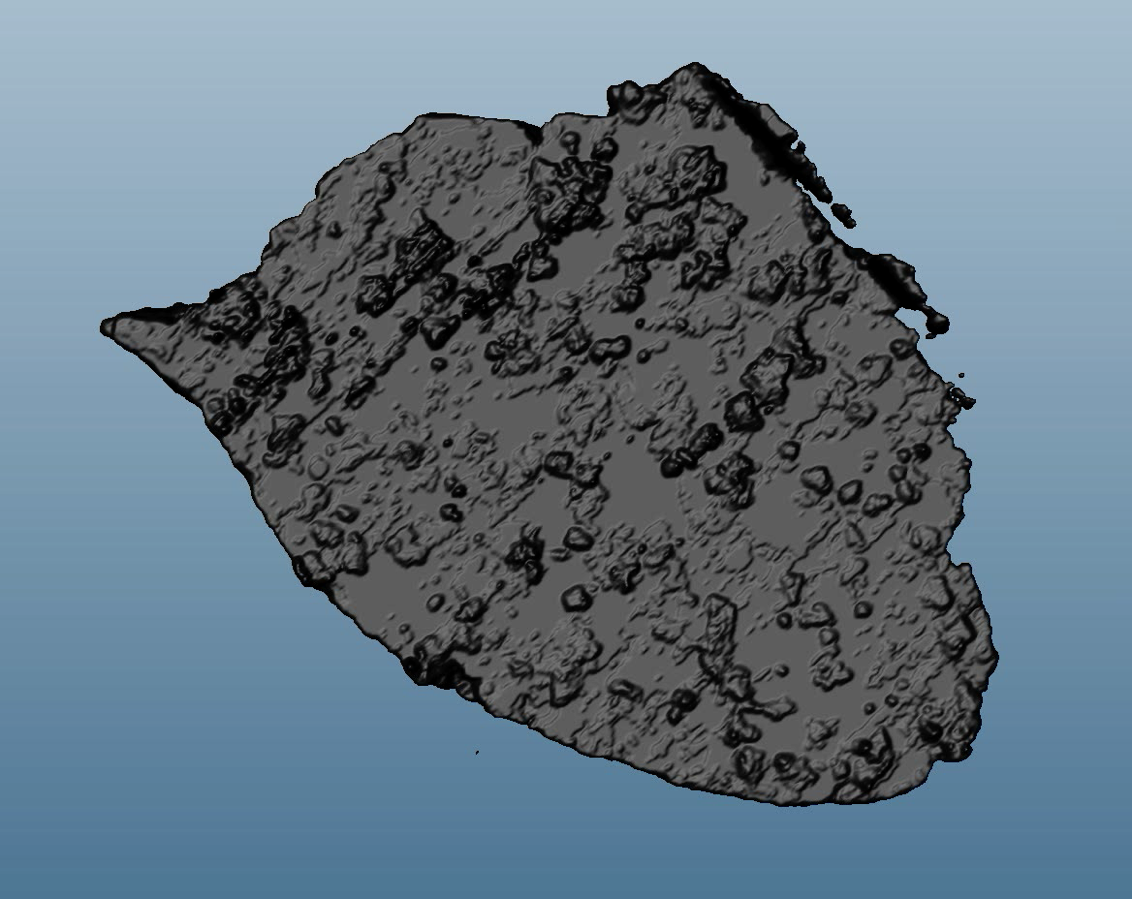 |
| --- | --- |

Figure S4. (a) Representative μCT images of Hamburg fragment 0.59 g taken at 10.65 μm isotropic voxel. This small fragment shows the rich FeNi (brightest white, outlined red in lower panel) and Fe-Sulfide (slightly darker, outlined blue in lower panel) textures of this meteorite. Image intensity was adjusted to allow for visual differentiation of the FeNi and Fe-Sulfide inclusions. (b) Animation of μCT data.


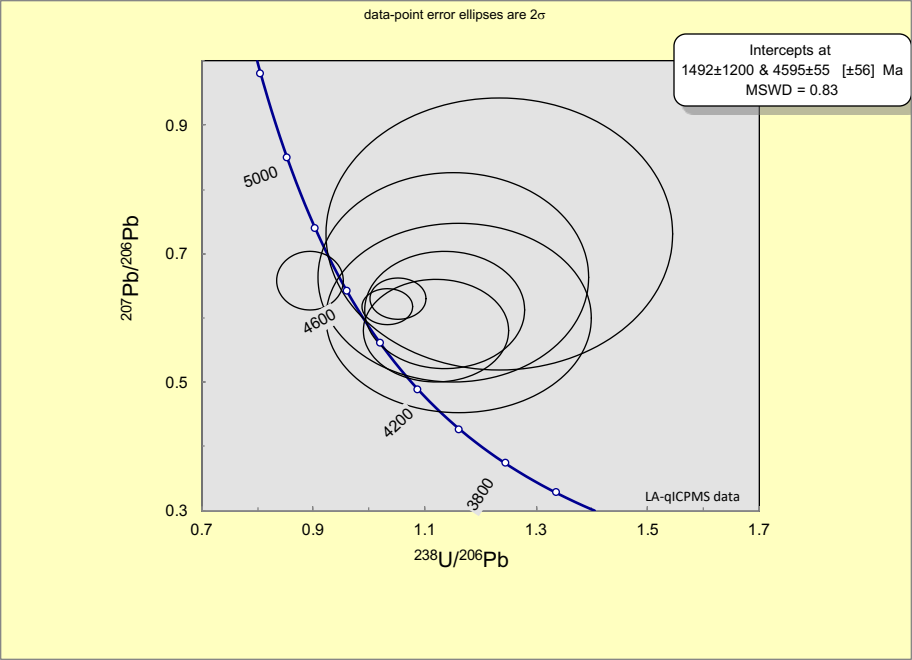

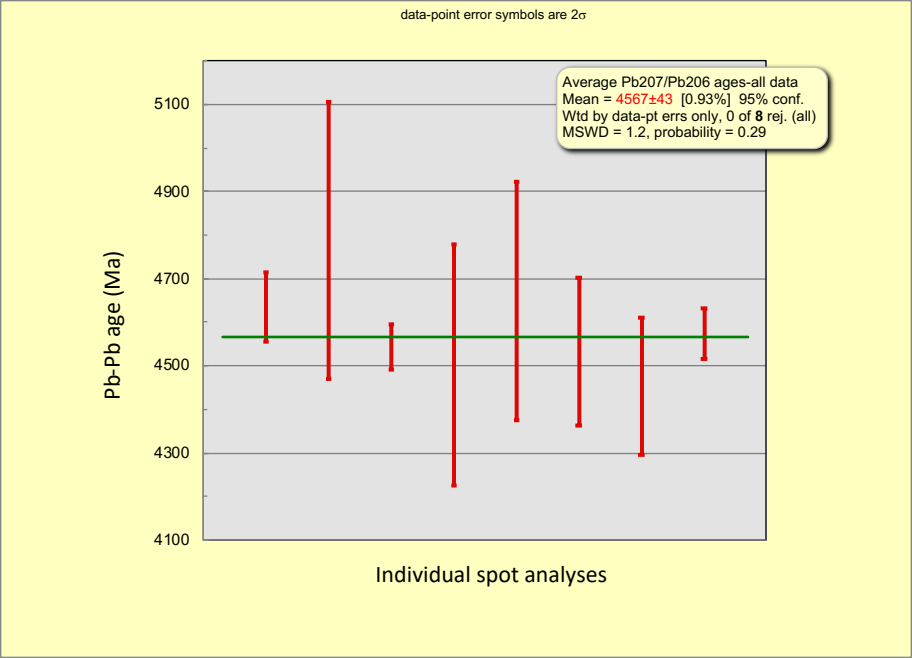


Figure S5. (a) Concordia diagram from Hamburg U-Pb LA-ICPMS data. (b) Pb-Pb LA-ICPMS data from Hamburg.


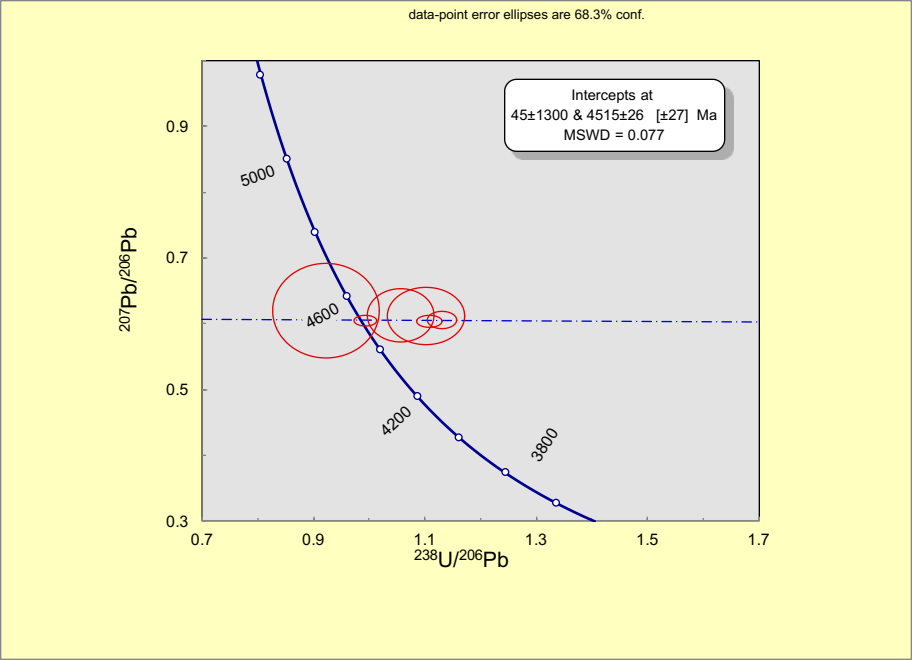

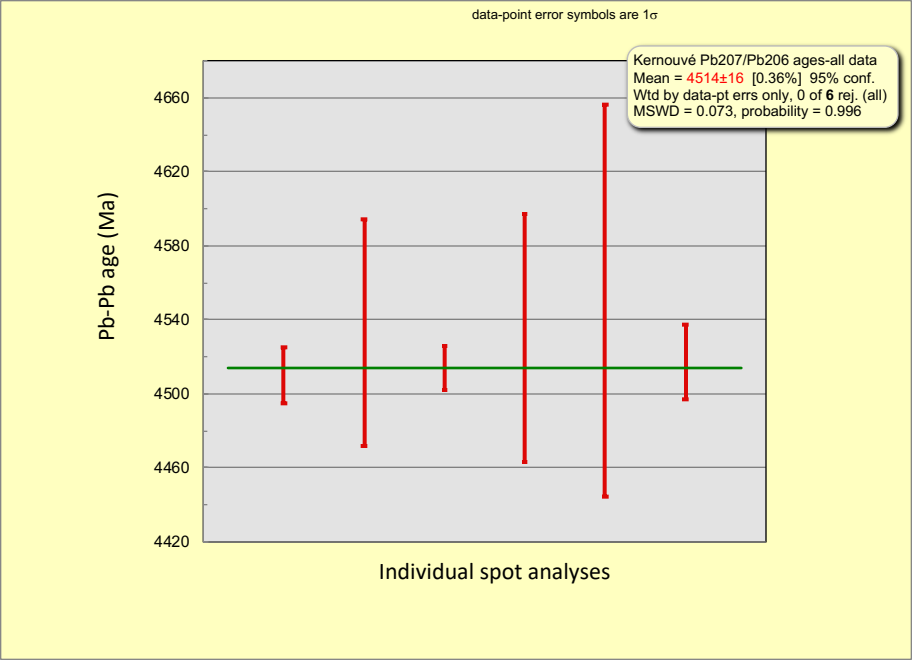
Figure S6. U-Pb and Pb-Pb data from Kernouvé for comparison to Hamburg. (a) Concordia diagram from Kernouvé U-Pb LA-ICPMS data. (b) Pb-Pb LA-ICPMS data from Kernouvé.


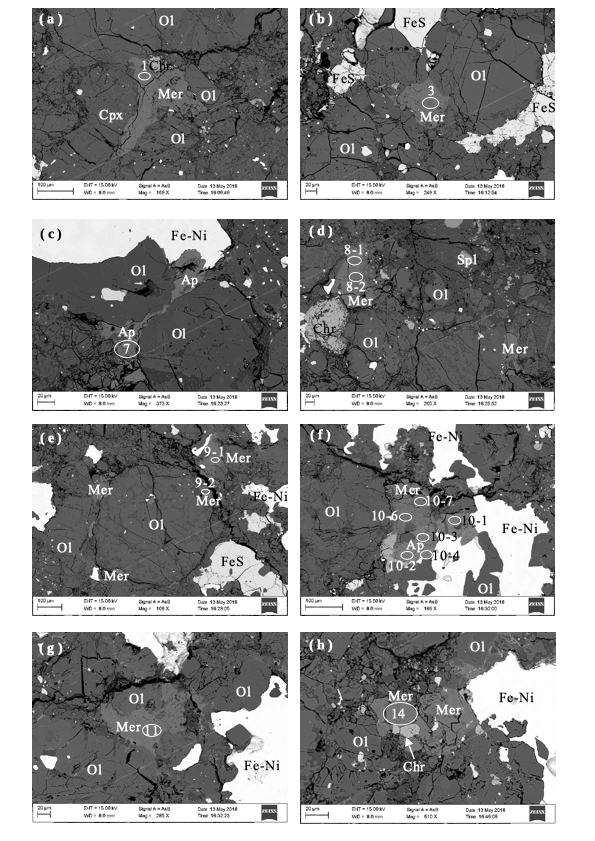


Fig. S7. SIMS analyses spots of the phosphate minerals in Hamburg L4 chondrite with their petrographic context.

**REFERENCES USED IN SUPPLEMENT**

Blackburn T., Alexander C. M. O., Carlson R., and Elkins-Tanton L. T. 2017. The accretion and impact history of the ordinary chondrite parent bodies. *Geochimica et Cosmochimica Acta* 200:201–217.

Bouvier A., Blichert-Toft J., Moynier F., Vervoort J. D., and Albarède F. 2007. Pb–Pb dating constraints on the accretion and cooling history of chondrites. *Geochimica et Cosmochimica Acta* 71:1583–1604.

Britt D. T., and Consolmagno G. J. 2003. Stony meteorite porosities and densities: A review of the data through 2001. *Meteoritics & Planetary Science* 38:1161–1180.

Camacho A., Baadsgaard H., Davis D. W., and Cerny P. 2012. Radiogenic isotope systematics of the Tanco and Silverleaf granitic pegmatites, Winnipeg river pegmatite district, Manitoba. *The Canadian Mineralogist* 50:1775–1792.

Cassata W. S., Cohen B. E., Mark D. F., Trappitsch R., Crow C. A., Wimpenny J., Lee M. R., and Smith C. L. 2018. Chronology of martian breccia NWA 7034 and the formation of the martian crustal dichotomy. *Science Advances* 4:eaap8306.

Davis D. W. 1982. Optimum linear regression and error estimation applied to U–Pb data. *Canadian Journal of Earth Sciences* 19:2141–2149.

Fries M., and Fries J. 2010a. Partly cloudy with a chance of chondrites –studying meteorite falls using Doppler weather radar. *Lunar and Planetary Science* 41:#1179.

Fries M., and Fries J. 2010b. Doppler weather radar as a meteorite recovery tool. *Meteoritics and Planetary Science* 45:1476–1487.

Fries M. D., and Fries J. A. 2010c. Doppler weather radar observations of the 14 April 2010 Southwest Wisconsin meteorite fall. *Meteoritics and Planetary Science Supplement* 45:#5365.

Fries M., Fries J., and Schaefer J. 2011. A probable unexplored meteorite fall found in archived weather radar data. *Lunar and Planetary Science* 42:#1130.

Fries M. D., Matson R., Schaefer J., and Fries J. A. 2012. Using weather radar data for rapid meteorite recovery: The “Sutter’s Mill” meteorite fall. *Meteoritics and Planetary Science Supplement* 47:#5388.

Fries M., Matson R., Schaefer J., Fries J., and Hankey M. 2013. Faster recovery, better science: meteorite fall events detected with weather radars and seismometers in 2012. *Lunar and Planetary Science* 44:#2935.

Fries M., Fries J., Hankey M., and Matson R. 2016. Meteorite falls observed in U.S. weather radar data in 2015 and 2016 (to date). *Meteoritics & Planetary Science, Supplement* 51:#6536.

Gauthreaux S. A., Belser C. G., Jr. S. A. G., and Belser C. G. 1998. Displays of bird movements on the WSR-88D: patterns and quantification. *Weather and Forecasting* 13:453–464.

Goldmann A., Brennecka G., Noordmann J., Weyer S., and Wadhwa M. 2015. The uranium isotopic composition of the Earth and the Solar System. *Geochimica et Cosmochimica Acta* 148:145–158.

Göpel C., Manhès G., and Allègre C. J. 1994. U-Pb systematics of phosphates from equilibrated ordinary chondrites. *Earth and Planetary Science Letters* 121:153–171.

Jaffey A. H., Flynn K. F., Glendenin L. E., Bentley W. C., and Essling A. M. 1971. Precision Measurement of Half-Lives and Specific Activities of ^235^U and ^238^U. *Physical Review B* 4:1889–1906.

Jarosewich E. 1990. Chemical analyses of meteorites: A compilation of stony and iron meteorite analyses. *Meteoritics* 25:323–337.

Leya I., and Masarik J. 2009. Cosmogenic nuclides in stony meteorites revisited. *Meteoritics & Planetary Science* 44:1061–1086.

Li Q.-L., Li X.-H., Wu F.-Y., Yin Q.-Z., Ye H.-M., Liu Y., Tang G.-Q., and Zhang C.-L. 2012. In-situ SIMS U–Pb dating of phanerozoic apatite with low U and high common Pb. *Gondwana Research* 21:745–756.

Ludwig K. R. 2003. User’s manual for Isoplot 3.00 - a geochronological toolkit for Microsoft Excel. *Berkeley Geochronology Center Special Publication*.

Mason B. 1979. Cosmochemistry part 1. Meteorites. In *Data of Geochemistry*, edited by Fleischer F. Geological Survey Professional Paper 440-B-1. pp. B1–B132.

Miller M. F. 2002. Isotopic fractionation and the quantification of ^17^O anomalies in the oxygen three-isotope system: an appraisal and geochemical significance. *Geochimica et Cosmochimica Acta* 66:1881–1889.

Popova O. P. et al. 2013. Chelyabinsk airburst, damage assessment, meteorite recovery, and characterization. *Science* 342:1069–1073.

Pouchou J.-L., and Pichoir F. 1991. Quantitative analysis of homogeneous or stratified microvolumes applying the model “PAP.” In *Electron Probe Quantitation*. Boston, MA: Springer US. pp. 31–75.

Ruf A. et al. 2017. Previously unknown class of metalorganic compounds revealed in meteorites. *Proceedings of the National Academy of Sciences* 114:2819–2824.

Sano Y., Oyama T., Terada K., and Hidaka H. 1999. Ion microprobe U–Pb dating of apatite. *Chemical Geology* 153:249–258.

Schindelin J. et al. 2012. Fiji: An open-source platform for biological-image analysis. *Nature Methods* 9:676–682.

Schmaljohann H., Liechti F., Bächler E., Steuri T., and Bruderer B. 2008. Quantification of bird migration by radar - a detection probability problem. *Ibis* 150:342–355.

Schmitt-Kopplin P., Gabelica Z., Gougeon R. D., Fekete A., Kanawati B., Harir M., Gebefuegi I., Eckel G., and Hertkorn N. 2010. High molecular diversity of extraterrestrial organic matter in Murchison meteorite revealed 40 years after its fall. *Proceedings of the National Academy of Sciences* 107:2763–2768.

Schoene B., Crowley J. L., Condon D. J., Schmitz M. D., and Bowring S. A. 2006. Reassessing the uranium decay constants for geochronology using ID-TIMS U–Pb data. *Geochimica et Cosmochimica Acta* 70:426–445.

Sharp Z. D. 1990. A laser-based microanalytical method for the in situ determination of oxygen isotope ratios of silicates and oxides. *Geochimica et Cosmochimica Acta* 54:1353–1357.

Shields W. R., Murphy T. J., Catanzaro E. J., and Garner E. L. 1966. Absolute isotopic abundance ratios and the atomic weight of a reference sample of chromium. *Journal of Research of the National Bureau of Standards Section A: Physics and Chemistry* 70A:193.

Shinotsuka K., Hidaka H., and Ebihara M. 1995. Detailed abundances of rare earth elements, thorium and uranium in chondritic meteorites: An ICP-MS study. *Meteoritics* 30:694–699.

Tatsumoto M., Knight R. J., and Allegre C. J. 1973. Time differences in the formation of meteorites as determined from the ratio of lead-207 to lead-206. *Science* 180:1279–1283.

Tera F., and Wasserburg G. J. 1972a. U-Th-Pb systematics in three Apollo 14 basalts and the problem of initial Pb in lunar rocks. *Earth and Planetary Science Letters* 14:281–304.

Tera F., and Wasserburg G. J. 1972b. U-Th-Pb systematics in lunar highland samples from the Luna 20 and Apollo 16 missions. *Earth and Planetary Science Letters* 17:36–51.

Thomson S. N., Gehrels G. E., Ruiz J., and Buchwaldt R. 2012. Routine low-damage apatite U-Pb dating using laser ablation-multicollector-ICPMS. *Geochemistry, Geophysics, Geosystems* 13:#Q0AA21 (23 pp).

Trotter J. A., and Eggins S. M. 2006. Chemical systematics of conodont apatite determined by laser ablation ICPMS. *Chemical Geology* 233:196–216.

Tziotis D., Hertkorn N., and Schmitt-Kopplin P. 2011. Kendrick-analogous network visualisation of ion cyclotron resonance Fourier transform mass spectra: improved options for the assignment of elemental compositions and the classification of organic molecular complexity. *European Journal of Mass Spectrometry* 17:415–421.

Wetherill G. W. 1956. Discordant uranium-lead ages, I. *Transactions, American Geophysical Union* 37:320.

Wilkison S. L., McCoy T. J., McCamant J. E., Robinson M. S., and Britt D. T. 2003. Porosity and density of ordinary chondrites: Clues to the formation of friable and porous ordinary chondrites. *Meteoritics & Planetary Science* 38:1533–1546.

Yamakawa A., Yamashita K., Makishima A., and Nakamura E. 2009. Chemical separation and mass spectrometry of Cr, Fe, Ni, Zn, and Cu in terrestrial and extraterrestrial materials using thermal ionization mass spectrometry. *Analytical Chemistry* 81:9787–9794.

Yin Q.-Z., Zhou Q., Li Q.-L., Li X.-H., Liu Y., Tang G.-Q., Krot A. N., and Jenniskens P. 2014. Records of the Moon-forming impact and the 470 Ma disruption of the L chondrite parent body in the asteroid belt from U-Pb apatite ages of Novato (L6). *Meteoritics & Planetary Science* 49:1426–1439.

Zhang A.-C., Li Q.-L., Yurimoto H., Sakamoto N., Li X.-H., Hu S., Lin Y.-T., and Wang R.-C. 2016. Young asteroidal fluid activity revealed by absolute age from apatite in carbonaceous chondrite. *Nature Communications* 7:12844.

Zhou Q., Yin Q.-Z., Shearer C. K., Li X.-H., Li Q.-L., Liu Y., Tang G.-Q., and Li C.-L. 2018. U-Pb and Pb-Pb apatite ages for Antarctic achondrite Graves Nunataks 06129. *Meteoritics & Planetary Science* 53:448–466.
